# Causal inference in survival analysis using longitudinal observational data: Sequential trials and marginal structural models

**DOI:** 10.1002/sim.9718

**Published:** 2023-04-22

**Authors:** Ruth H. Keogh, Jon Michael Gran, Shaun R. Seaman, Gwyneth Davies, Stijn Vansteelandt

**Affiliations:** ^1^ Department of Medical Statistics and Centre for Statistical Methodology London School of Hygiene and Tropical Medicine Keppel Street London WC1E 7HT UK; ^2^ Oslo Centre for Biostatistics and Epidemiology, Department of Biostatistics, Institute of Basic Medical Sciences University of Oslo P.O. Box 1122 Blindern Oslo 0317 Norway; ^3^ MRC Biostatistics Unit University of Cambridge East Forvie Building, Forvie Site, Robinson Way Cambridge CB2 0SR UK; ^4^ Population, Policy and Practice Research and Teaching Department, UCL Great Ormond Street Institute of Child Health University College London WC1N 1EH London UK; ^5^ Department of Applied Mathematics, Computer Science and Statistics Ghent University 9000 Ghent Belgium

**Keywords:** cystic fibrosis, inverse probability weighting, marginal structural model, registries, sequential trials, survival, target trials, time‐dependent confounding

## Abstract

Longitudinal observational data on patients can be used to investigate causal effects of time‐varying treatments on time‐to‐event outcomes. Several methods have been developed for estimating such effects by controlling for the time‐dependent confounding that typically occurs. The most commonly used is marginal structural models (MSM) estimated using inverse probability of treatment weights (IPTW) (MSM‐IPTW). An alternative, the sequential trials approach, is increasingly popular, and involves creating a sequence of “trials” from new time origins and comparing treatment initiators and non‐initiators. Individuals are censored when they deviate from their treatment assignment at the start of each “trial” (initiator or noninitiator), which is accounted for using inverse probability of censoring weights. The analysis uses data combined across trials. We show that the sequential trials approach can estimate the parameters of a particular MSM. The causal estimand that we focus on is the marginal risk difference between the sustained treatment strategies of “always treat” vs “never treat.” We compare how the sequential trials approach and MSM‐IPTW estimate this estimand, and discuss their assumptions and how data are used differently. The performance of the two approaches is compared in a simulation study. The sequential trials approach, which tends to involve less extreme weights than MSM‐IPTW, results in greater efficiency for estimating the marginal risk difference at most follow‐up times, but this can, in certain scenarios, be reversed at later time points and relies on modelling assumptions. We apply the methods to longitudinal observational data from the UK Cystic Fibrosis Registry to estimate the effect of dornase alfa on survival.

## 1 INTRODUCTION

Longitudinal observational data on patients can allow for the estimation of treatment effects over long follow‐up periods and in diverse populations. A key task when aiming to estimate causal effects from observational data is to account for confounding of the treatment‐outcome association. With longitudinal data on treatment and outcome, the treatment‐outcome association may be subject to so‐called time‐dependent confounding. Such confounding is present when there are time‐dependent covariates affected by past treatment that also affect later treatment use and outcome. It presents particular challenges for estimation of the effects of longitudinal treatment regimes, such as those that involve comparing sustained treatment or no treatment over time. Over the last two decades a large statistical literature has built up on methods for estimating causal treatment effects in the presence of time‐dependent confounding, and their use in practice is becoming more widespread.

In this paper we focus on estimating the causal effects of longitudinal treatment regimes on a time‐to‐event outcome, using longitudinal observational data on treatment and covariates obtained at approximately regular visits, alongside the time‐to‐event information. When there is time‐dependent confounding, standard methods for survival analysis, such as Cox regression with adjustment for baseline or time‐updated covariates, do not generally enable estimation of such causal effects directly. Several methods have been described for estimating the causal effects of longitudinal treatment regimes on time‐to‐event outcomes. Marginal structural models (MSMs) estimated using inverse probability of treatment weighting (IPTW) were introduced by Robins et al^1^ and extended to survival outcomes by Hernan et al^2^ through marginal structural Cox models. A review by Clare et al in 2019^3^ found this approach to be by far the most commonly used in practice, and we refer to this as the MSM‐IPTW approach. An alternative but related approach, which we refer to as the “sequential trials” approach, was described by Hernan et al in 2008^4^ and Gran et al in 2010.^5^ With the sequential trials approach, artificial “trials” are created from a sequence of new time origins which could, for example, be defined by study visits at which new information is recorded for each individual who remains under observation. At each time origin individuals are divided into those who have just initiated the treatment under investigation and those who have not yet initiated the treatment. Within each trial, individuals are artificially censored at the time at which their treatment status deviates from what it was at the time origin, if such deviation occurs. Inverse probability of censoring weighting is used to account for dependence of this artificial censoring on time‐dependent characteristics. The overall effects of sustained treatment versus no treatment, starting from each time origin, can then be estimated using, for example, weighted pooled logistic or Cox regression. The sequential trials approach was originally proposed as a simple way of making efficient use of longitudinal observational data, as it enables use of a larger sample size than if an artificial trial were formed from a single time origin.^4^ Hernan et al^4^ did not discuss time‐dependent confounding directly, but focused on the more general concept of emulating a sequence of hypothetical randomized trials from observational data. Their initial focus was on estimation of intention‐to‐treat effects, which does not require censoring of individuals when they deviate from their initial treatment status. However, they also considered estimation of per protocol effects, which focus on sustained treatment vs no treatment, using censoring and weighting, and they referred to these as “adherence adjusted” effects. Gran et al^5^ focused on per protocol effects, and assumed that individuals who started treatment always continued it, but that noninitiators of treatment at the start of each trial could start treatment at later time, which they addressed using censoring and weighting. In this paper we focus on per protocol effects, that is, on sustained treatment strategies.

The sequential trials approach is closely related to several other methods for estimation of treatment effects using longitudinal data. Thomas et al^6^ reviewed such methods, referring to them collectively as “longitudinal matching methods.” In addition to the sequential trials approach, these include the sequential stratification approaches suggested by Schaubel et al in 2006 and 2009^7^, ^8^ and considered in a number of subsequent papers such as that of Gong and Schaubel.^9^ In their discussion, Thomas et al^6^ noted that most methods they reviewed, including the sequential trials approach, had not been connected with the counterfactual outcomes framework for defining causal estimands of interest. They did not include MSM‐IPTW in their review, but implied in the discussion that the MSM‐IPTW and sequential trials approaches address different questions. In this paper, we show how the MSM‐IPTW approach and the sequential trials approach can be used to estimate the same causal estimand for sustained treatment regimes.

There have been several applications of the sequential trials approach. Danaei et al^10^ applied it to electronic health records data to estimate the effect of statins on occurrence of coronary heart disease. Clark et al^11^ studied whether onset of impaired sleep affects health related behavior. Bhupathiraju et al^12^ investigated the effect of hormone therapy use on chronic disease risk in the Nurses' Health Study. Caniglia et al^13^ estimated the effects of statin use on risk of dementia up to 10 years using data from a prospective cohort study. Both Danaei et al^10^ and Caniglia et al^13^ considered per protocol effects, while the other examples referenced above focused on intention‐to‐treat effects. Some papers have also used the sequential trials approach alongside other methods and compared the results empirically. Suttorp et al^14^ studied the effect of high dose of a particular agent used in kidney disease patients on all‐cause mortality with both a sequential trials approach and MSM‐IPTW using data from a longitudinal cohort. In their review paper, Thomas et al^6^ illustrated the sequential trials approach together with sequential stratification and time‐dependent propensity score matching to study the effect of statins on cardiovascular outcomes using the Framingham Offspring cohort. Several papers in recent years have advocated the benefits of explicitly emulating a “target trial”—the hypothetical randomized controlled trial that one would ideally like to conduct—in investigations of causal effects using observational data.^15^, ^16^, ^17^, ^18^, ^19^ The use of target trials provides a structured framework that guides the steps of an investigation, with the target trial protocol including specification of eligibility criteria, time origin, and the causal estimand of interest. This framework has been used in conjunction with the sequential trials approach, for example by Caniglia et al.^13^

In most applications of the sequential trials approach the target causal parameters have been informally stated using the language of emulating hypothetical randomized trials. However, a more formal description of such parameters using counterfactuals has been lacking. Gran and Aalen^20^ highlighted the need for further work to establish the causal estimands that were being estimated in the sequential trials approach. This is important both to clarify the interpretation and to inform simulation studies to study the properties of the estimators. Karim et al^21^ compared the sequential trials approach with MSM‐IPTW based on Cox models using simulations, but did not account for the fact that in their implementation the two analyses were used to estimate different quantities, which were therefore not directly comparable.^20^ The aim of this paper is to establish connections between the MSM‐IPTW approach and the sequential trials approach, and outline where they can both be used to estimate the same causal estimand, focusing on a marginal risk difference. We show that the causal parameter estimated from the sequential trials approach can be described in terms of the parameters of a particular MSM using the counterfactual framework. We also establish more broadly what estimands the MSM‐IPTW and sequential trials approaches are suitable for identifying, what assumptions are needed, and how the data are used differently. We compare the MSM‐IPTW and sequential trials approaches in a simulation study, in which we assess their relative efficiency for estimation of the same quantities.

In most of the literature on the sequential trials approach, including the papers of Hernan et al^4^ and Gran et al,^5^ the primary focus has been on estimation of hazard ratios using Cox proportional hazard models, or the approximately equivalent pooled logistic regression model. This has traditionally also been the case for the MSM‐IPTW for time‐to‐event outcomes. The Aalen additive hazard models for hazard differences have also become increasingly popular, both to avoid the proportional hazards assumption and due to their useful property of being collapsible.^22^ However, hazard ratios, and generally also hazard differences, have been shown not to have a straightforward causal interpretation.^23^, ^24^, ^25^ By focusing on the marginal risk difference we avoid this issue. While other studies^4^, ^13^, ^18^, ^26^ have presented estimated survival curves under the treated and untreated regimes, and corresponding risk differences, it has not been described in detail how such survival curves have been or can be obtained, or what population they refer to. In this paper we demonstrate how our causal estimand of interest, a marginal risk difference, can be estimated under both the sequential trials and MSM‐IPTW approaches by using an empirical standardization procedure. This is similar to the standardization approach described by Young et al.^27^ We consider both proportional and additive hazards models and our simulation comparison of the two approaches makes use of the properties of additive hazards models.^28^

The paper is organized as follows. In Section 2 we describe a motivating example, in which the aim is to estimate the effect of long term use of the treatment dornase alfa on the composite outcome of death or transplant for people with cystic fibrosis (CF), using longitudinal data from the UK Cystic Fibrosis Registry.^29^ This section also sets out the notation used throughout the paper. In Section 3 we define the causal estimand of interest, which is a marginal risk difference. The estimand is defined using counterfactual notation. In Section 4 we consider how the causal estimand can be identified using the longitudinal observational data, and outline estimation using the MSM‐IPTW approach and the sequential trials approach. We show how the sequential trials approach can also be understood as fitting a particular MSM. In Section 5 we discuss in detail the similarities and differences between the two approaches. The two approaches are then compared in a simulation study in Section 6, and applied to the motivating example in Section 7. Accompanying R code for performing the simulation is provided at https://github.com/ruthkeogh/sequential_trials. We conclude with a discussion in Section 8.

## 2 MOTIVATING EXAMPLE AND DATA STRUCTURE

### 2.1 Investigating treatment effects in CF

As a motivating example we will investigate the long‐term impacts of a treatment used in CF on the composite time‐to‐event outcome of death or transplant. CF is an inherited, chronic, progressive condition affecting around 10 500 individuals in the United Kingdom and over 100 000 worldwide.^30^, ^31^, ^32^, ^33^ Survival in CF has improved considerably over recent decades and the estimated median survival age in the UK is 51.6 for males and 45.7 for females.^30^, ^34^, ^35^

One of the most common consequences of CF is a build up of mucus in the lungs, which leads to an increased prevalence of bacterial growth in the airways and a decline in lung function.^36^ Therefore, it is common for people with CF to routinely use aerosolized mucoactive agents, which help to break down the layer of mucus in the lungs making clearance easier. Recombinant human deoxyribonuclease, commonly known as dornase alfa (DNase), is one such mucolytic treatment which was authorized for use in 1994. DNase is the most commonly used treatment for the pulmonary consequences of CF in the United Kingdom, used by 67.6% of CF patients in 2019.^30^ It is a nebulized treatment, administered daily on a long term basis. The efficacy of DNase for health outcomes in CF has been studied in several randomized controlled trials.^37^ Most trials had short‐term follow‐up and focused on the impact of DNase on lung function. Seven trials from the 1990s investigated mortality as a secondary outcome but follow‐up was short and findings inconclusive; a meta‐analysis based on these studies gave a mortality risk ratio estimate of 1.70 (95% CI 0.70‐4.14) comparing users vs nonusers.^37^

The effect of DNase use on lung function and requirement for intravenous antibiotics has previously been investigated using observational data from the UK Cystic Fibrosis Registry.^38^, ^39^ We now use the UK Cystic Fibrosis Registry to investigate the impact of DNase on the composite outcome of death or transplant. The majority of transplants in people with CF are lung transplants, however, we consider any transplant here. The UK Cystic Fibrosis Registry is a national, secure database sponsored and managed by the Cystic Fibrosis Trust.^29^ It was established in 1995 and records demographic data and longitudinal health data on nearly all people with CF in the United Kingdom, to date capturing data on over 12 000 individuals. Data are collected in a standardized way at designated (approximately) annual visits on over 250 variables in several domains, and have been recorded using a centralised database since 2007. In this study we use data from 2008 to 2018, plus some data on prior years to define eligibility for inclusion in the analysis according to criteria outlined below. At each annual visit it is recorded whether or not an individual had been using DNase in the past year. We identified potential confounders of the association between DNase use and death or transplant based on expert clinical input, and these include both time‐fixed and time‐dependent variables—see Section 2.2. We focus on the impact of initiating and continuing use of DNase on the risk of death or transplant up to 11 years of follow‐up compared with not using DNase.

### 2.2 Notation and data structure

We assume a setting in which individuals are observed at regular “visits” (data collection times) over a particular calendar period (2008‐2018 in the example), reflecting the type of data that arise in our motivating example and in similar data sources. Let k=0 denote the start of follow‐up for a given patient, which we define in our context as the first time in the analysis dataset at which they are first observed to meet some specified eligibility criteria and at which they could initiate a treatment strategy of interest. This is discussed further below and in Table 1. Time k=0 can be at different points in calendar time for different individuals.

**TABLE 1:** Summary of the protocol of a target trial for studying the effect of DNase use on survival in people with cystic fibrosis (CF), and summary of how the target trial is emulated using UK CF Registry data.

Protocol component	Target trial	Emulation of the target trial using UK CF Registry data
Eligibility criteria	People with CF aged 12 or older and who have not used DNase for at least 3 years.	The target trial is emulated using data on people who were included in the UK CF registry between 2008 and 2018. The emulation is performed using the MSM‐IPTW and sequential trials approaches. At a given annual visit from 2008 to 2017, a person is defined as meeting the trial emulation eligibility criteria if they meet the following conditions: aged 12 or older; have at least three preceding visits, at least one in the prior 18 months, and were recorded as not using DNase at each of those visits; have observed measurements of FEV1% and BMI *z*‐score at the current and previous visits. The first visit at which the trial emulation eligibility criteria are met in the available data is denoted k=0 and subsequent visits times are k=1,2,…. The maximum number of visits is 11, with k=10 corresponding to 2018.
Treatment strategies	(i) Do not start using DNase during follow‐up. (ii) Start using DNase and continue to use it throughout follow‐up.	The treatment strategies of interest are as in the target trial. However, in the observational data individuals do not necessarily follow either of these strategies.
Assignment procedures	Patients randomly assigned to either treatment strategy. Patients and their health care teams are aware of the patient's treatment status.	At each visit an individual is a DNase user or non‐user, and individuals can switch on or off treatment. Treatment use is assumed to be informed by the following covariates, which are also assumed to affect the outcome: sex, genotype class, age, FEV 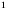 %, BMI z‐score, chronic infection with *Pseudomonas aeruginosa* or use of nebulized antibiotics, infection with *Staphylococcus aureus* or MRSA, infection with *Burkholderia cepacia*, infection with *Non‐tuberculous mycobacteria* (NTM), CF‐related diabetes, pancreatic insufficiency, number of days on intravenous (IV) antibiotics at home or in hospital, other hospitalization (yes or no), use of other mucoactive treatments (hypertonic saline, mannitol or acetylcysteine), use of oxygen therapy, use of CFTR modulators (ivacaftor, lumacaftor/ivacaftor, or tezacaftor/ivacaftor), and past DNase use. Patients and their health care teams are aware of the patient's treatment status.
Follow‐up period	Starts at randomization and ends at death or transplant, loss‐to follow‐up, or 11 years of follow‐up, whichever occurs first.	MSM‐IPTW approach: Follow‐up starts at k=0 and ends at death or transplant, loss‐to follow‐up, or the end of 2018, whichever occurs first.
		Sequential trials approach: A trial is formed from each visit k=0,1,…. Trial k includes individuals meeting the trial emulation eligibility criteria at visit k (k=0,1,...,9). In trial k follow‐up starts at time k and ends at switching away from the treatment status at visit k, death or transplant, loss to follow‐up, or the end of 2018, whichever occurs first.
Outcome	Death or transplant.	As in the target trial.
Causal contrasts	Per protocol treatment effect. Marginal survival curves up to 11 years under the two treatment strategies, and corresponding marginal risk differences. For the marginal probabilities, the population of interest is individuals meeting the target trial eligibility criteria in 2018.	The causal contrasts of interest are as in the target trial. Estimation is using the MSM‐IPTW approach and the sequential trials approach—see Section 4. The emulation of the target trial makes use of data from 2008 to 2018, however, for the marginal probabilities in the causal estimand, we target the population meeting the trial emulation eligibility criteria in 2018.

Information on treatment status and other covariates is observed at visits k=0,1,…. Without loss of generality, we assume that visit k occurs at time k, for k≥0. We consider a binary treatment A (treatment or control). At each visit k we observe binary treatment status $A_{k}$ and a set of time‐dependent covariates $L_{k}$. A bar over a time‐dependent variable indicates the history, that is ${\bar{A}}_{k}=\{A_{0},A_{1},\ldots ,A_{k}\}$ and ${\bar{L}}_{k}=\{L_{0},L_{1},\ldots ,L_{k}\}$. An underline indicates the future, so that ${\underline{A}}_{k}=\{A_{k},A_{k+1},\ldots \}$ denotes treatment status from time k onwards. In a slight abuse of standard notation we let ⌊k⌋ denote the time of the most recent visit *before* time k (so ⌊k⌋ is always <k), and ${\bar{A}}_{\left\lfloor k\right\rfloor }$ denotes the treatment pattern up to the most recent visit prior to k. Individuals are followed up from time k=0 until the time of the event of interest (death or transplant) or the time of censoring, whichever occurs first. Censoring could be due to loss‐to‐follow‐up or administrative censoring at the date of data extraction.

**FIGURE 1:**
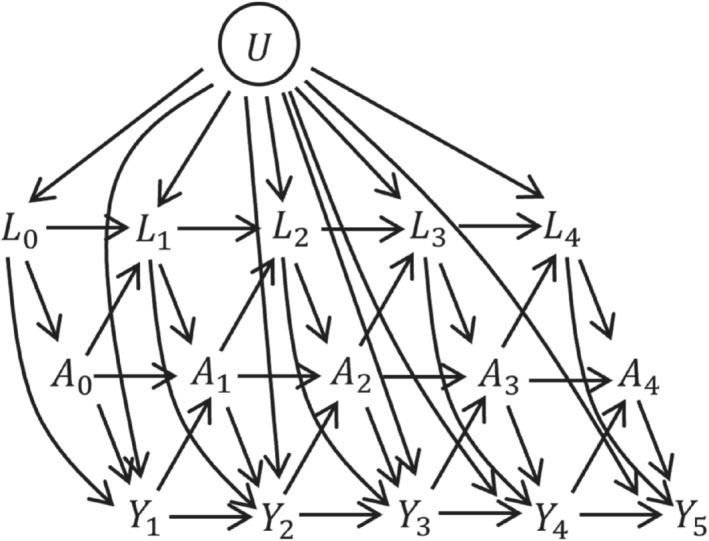
Directed acyclic graph (DAG) illustrating relationships between treatment A, time‐dependent covariates L, discrete time outcome Y, and unmeasured covariates U. Time‐fixed covariates Z are omitted from the diagram but are assumed to potentially affect all other variables.

The assumed data structure is illustrated in the directed acyclic graph (DAG) in Figure 1, using a discrete‐time setting where $Y_{k}=I(k-1<T\leq k)$ is an indicator of whether the event occurs between visits k−1 and k. One can imagine extending the DAG by adding a series of small time intervals between each visit, at which events are observed (but not A or L, which are assumed constant between visits). As the time intervals become very small we approach the continuous time setting. From the DAG, we can see that $L_{k}$ are time‐dependent confounders. Time‐dependent confounding occurs when there are time‐dependent covariates that predict subsequent treatment use, are affected by earlier treatment, and affect the outcome through pathways that are not just through subsequent treatment. The DAG also includes a variable U, which has direct effects on $L_{k}$ and $Y_{k}$ but not on $A_{k}$. U is an unmeasured individual frailty and we include it because it is realistic that such individual frailty effects exist in practice. Because U is not a confounder of the association between $A_{k}$ and $Y_{k}$ (after controlling for the observed confounders), the fact that it is unmeasured does not affect our ability to estimate causal effects of treatments. The DAG represents the assumed data structure and informs which variables, measured at what time points, are confounders of the association between treatment at a given time point and the outcome. The DAG could be extended in various ways, for example, we could incorporate long term effects of L on A and vice versa by adding arrows from $L_{k}$ to $A_{k+1}$ and from $A_{k}$ to $L_{k+2}$. Long term effects of A and L on survival could also be added, for example by adding arrows from $L_{k}$ and $A_{k}$ to $Y_{k+2}$. The DAG could also include time‐fixed confounders, which we denote as Z.

In the UK CF Registry data, DNase use ($A_{k}$) is recorded at each visit and refers to whether the patient has been prescribed DNase over the past year. The time‐dependent confounders ($L_{k}$) that we consider are: age (in years), lung function measured using forced expiratory volume in 1 second as percentage predicted (FEV
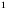
%), body mass index (BMI) z‐score, chronic infection with *Pseudomonas aeruginosa* or use of nebulized antibiotics, infection with *Staphylococcus aureus* or Methicillin‐resistant *Staphylococcus aureus* (MRSA), infection with *Burkholderia cepacia*, infection with *Non‐tuberculous mycobacteria* (NTM), CF‐related diabetes, pancreatic insufficiency, number of days on intravenous (IV) antibiotics at home or in hospital (categorized as 0, 1‐14, 15‐28, 29‐42, 43+), other hospitalization, use of other mucoactive treatments (hypertonic saline, mannitol or acetylcysteine), use of oxygen therapy, use of CFTR modulators (ivacaftor, lumacaftor/ivacaftor, or tezacaftor/ivacaftor). Of the time‐dependent variables, FEV
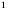
% and BMI are measured on the day of the visit, whereas all of the other variables refer to information in the time since the previous visit, that is, in the past year. We also include two time‐fixed covariates (Z): sex (male/female) and genotype class (a marker of the severity of the CF‐causing mutation—low, high, not assigned, missing).^40^ The covariates are binary yes/no variables except where indicated. For individuals who are not observed to have the event of interest (death or transplant) the censoring date was the earlier of December 31, 2018 and the date of the last visit plus 18 months.

## 3 THE CAUSAL ESTIMAND

### 3.1 The general case

In this section we specify the causal estimand, meaning the quantity that we wish to estimate. We consider the estimand in general terms first, and then in the context of our example introduced in Section 2. Our interest lies in comparing outcomes under two treatment strategies: (1) to start and continue treatment (to the time horizon of interest); (2) not to start treatment up to the time horizon of interest. We refer to strategy (1) as “always treated” and strategy (2) as “never treated.” The time scale on which the causal estimand is defined is denoted t, with t=0 denoting the time at which an individual could initiate strategy (1) or (2). Let us define $T^{{\underline{a}}_{0}=1}$ as the counterfactual event time had an individual received the treatment from t=0 onwards (“always treated”) and $T^{{\underline{a}}_{0}=0}$ as the counterfactual event time had an individual not received the treatment from t=0 onwards (“never treated”). We can then define our causal estimands of interest as contrasts between functions of the distributions of $T^{{\underline{a}}_{0}=1}$ and $T^{{\underline{a}}_{0}=0}$.

Effects of treatments on time‐to‐event outcomes can be quantified in a number of ways. We let $h_{T^{{\underline{a}}_{0}}}(t)$ denote the counterfactual hazard at time t under the possibly counter‐to‐fact treatment strategy ${\underline{a}}_{0}$. A common estimand is the ratio of hazards between the treatment and control strategies, $h_{T^{{\underline{a}}_{0}=1}}(t)/h_{T^{{\underline{a}}_{0}=0}}(t)$. However, as hazard ratios do not have a straightforward causal interpretation, it is recommended to consider causal contrasts between risks.^23^, ^24^, ^25^ In this paper we will focus on the risk difference as the primary estimand of interest:

(1) $$\mathrm{Pr}(T^{{\underline{a}}_{0}=1}\leq \tau )-\mathrm{Pr}(T^{{\underline{a}}_{0}=0}\leq \tau )=\mathrm{Pr}(T^{{\underline{a}}_{0}=0}>\tau )-\mathrm{Pr}(T^{{\underline{a}}_{0}=1}>\tau ).$$

This risk difference contrasts the risk of the event by a time horizon τ, under the conditions of being “always treated” vs “never treated” up to time τ. There may be several time horizons of interest, that is, different values of τ, and we let $\tau_{\max}$ denote the maximum time horizon for which the risk difference is to be obtained. Alternative quantities with a causal interpretation are the risk ratio $\mathrm{Pr}(T^{{\underline{a}}_{0}=1}<\tau )/\mathrm{Pr}(T^{{\underline{a}}_{0}=0}<\tau )$, the survival ratio $\mathrm{Pr}(T^{{\underline{a}}_{0}=1}>\tau )/\mathrm{Pr}(T^{{\underline{a}}_{0}=0}>\tau )$ and contrasts between restricted mean survival times $E(\min(T^{{\underline{a}}_{0}},\tau ))$.^41^

The causal estimand in (1) corresponds to a per protocol effect because it involves comparisons between outcomes if treatment level a (a=0,1) were sustained from t=0 up to time τ. The estimand is expressed in terms of marginal probabilities (risks) and it is important to make clear what population the marginal probabilities refer to, for example, in terms of age and clinical characteristics. If the causal estimand were to be estimated from a randomized trial then t=0 would be the time of randomization and the population to which the marginal risks refer would be the population from which the trial participants were drawn.

The probabilities in (1) can be written as a function of the counterfactual hazard, which we make use of later for estimation:

(2) $$\mathrm{Pr}(T^{{\underline{a}}_{0}=a}>\tau )=\exp\left(-\int_{0}^{\tau }h_{T^{{\underline{a}}_{0}=a}}(t)dt\right).$$

The counterfactual hazard function could also be considered as a function of the covariate history at t=0, denoted V, $h_{T^{{\underline{a}}_{0}=a}}(t|V)$. In this case we can write

(3) $$\mathrm{Pr}(T^{{\underline{a}}_{0}=a}>\tau )=E_{V}\left\{\mathrm{Pr}(T^{{\underline{a}}_{0}=a}>\tau |V)\right\}=E_{V}\left\{\exp\left(-\int_{0}^{\tau }h_{T^{{\underline{a}}_{0}=a}}(t|V)dt\right)\right\},$$

where the expectation is over the distribution of V in the population of interest.

### 3.2 Application in the UK CF Registry: causal estimand

In our motivating example, “always treated” means sustained use of DNase for the follow‐up period of interest and “never treated” means not using DNase for the follow‐up period of interest. We are interested in risk differences up to 11 years of follow‐up (0<τ≤11), standardized to the underlying population of individuals meeting some specified eligibility criteria in 2018, this being the most recent year for which data were available for this study, and therefore considered the population of most relevance to the current CF population. The characteristics of this population could be summarised by their distribution of time‐fixed covariates Z and their history of time‐dependent variables L up to 2018.

The causal estimand can be linked to a target trial.^15^ Arguably a precise specification of the causal estimand using counterfactual notation, as in (1), encompasses all elements of the target trial. However, by specifying the target trial alongside the causal estimand, we can help ensure that our aims and findings are clear and accessible by different audiences. Table 1 outlines the components of the target trial for studying the effect of DNase on survival on people with CF, and summarizes how the target trial is emulated using the available data, following examples in the literature.^13^, ^18^ There are differences in the way that the MSM‐IPTW and sequential trials approaches use the observed data for the trial emulation, which is incorporated in the description of the trial emulation.

The target trial protocol specifies the population to which the causal estimand of interest refers, which is individuals meeting the target trial eligibility criteria in 2018. The eligibility criteria for the trial emulation using the UK CF Registry data (“trial emulation eligibility criteria”) extend the target trial eligibility criteria as they take into account features of the data and how the data are to be used in the analysis. The emulation of the trial makes use of data from 2008 to 2018. For the marginal probabilities in the causal estimand the population of interest is people meeting the trial emulation eligibility criteria in 2018. For the trial emulation we identify individuals meeting the trial emulation eligibility criteria at visits recorded in the UK CF Registry data between 2008 and 2017. To do this also requires data from three visits prior to 2008 to ascertain whether the person had been taking DNase in the three previous years. Individuals who may have used DNase in the more distant past were included to increase sample size and because it was considered reasonable that the effect of restarting DNase in this group would be similar to that for individuals who had never used DNase in the past. The trial emulation eligibility criteria also include that an individual is eligible at a given visit if they have at least three preceding visits, with the patient recorded as not using DNase at each of those visits, and with at least one visit being in the prior 18 months. To be eligible at a given visit, individuals were also required to have observed measurements of FEV
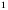
% and BMI z‐score at that visit and the previous visit.

## 4 ESTIMATION USING LONGITUDINAL OBSERVATIONAL DATA

### 4.1 Assumptions and MSMs

This section focuses on the estimation of the causal estimand in (1) using longitudinal observational data of the type described in Section 2.2 and as illustrated in the DAG in Figure 1. In the observational data, individuals who are untreated initially ($A_{0}=0$) can subsequently start treatment (ie, $A_{k}=1$ for some k≥1) and vice versa. As illustrated in the DAG in Figure 1, a change in a person's treatment status can depend on time‐dependent patient characteristics that also affect the outcome. Therefore, individuals who follow the treatment patterns of interest from time k=0 (ie, ${\underline{A}}_{0}=1$ [“always treated”] and ${\underline{A}}_{0}=0$ [“never treated”]), differ systematically in their characteristics at time k=0. Furthermore, some individuals follow other patterns of treatments, for example, $\left\{{\bar{A}}_{k-1}=0,{\underline{A}}_{k}=1\right\}$. To identify the causal estimand of interest from the observational data requires the four key assumptions of no interference, positivity, consistency, and conditional exchangeability (ie, no unmeasured confounding).^1^, ^42^, ^43^ Details on these assumptions are provided in Supplementary Material (Section 1). We did not depict censoring in the DAG, but it is also assumed that any censoring of individuals in the observational data is uninformative conditional on observed time‐fixed and time‐dependent covariates and treatments, and handling of censoring is discussed further below.

The causal estimand in (1) involves marginal risks, which we expressed in (2) in terms of the counterfactual hazard function. For estimation, we assume a particular model for these hazard functions, with the model specifying how the counterfactual hazard at a given time depends on the treatment history up to that time. Such models are referred to as MSMs. In the time‐to‐event setting, the MSM for a hazard is usually assumed to take the form of a Cox proportional hazards model^44^

(4) $$h_{T^{{\underline{a}}_{0}}}(t)=h_{0}(t)e^{g({\bar{a}}_{\left\lfloor t\right\rfloor };\beta_{A})},$$

where $h_{0}(t)$ is the baseline counterfactual hazard, ${\bar{a}}_{\left\lfloor t\right\rfloor }$ denotes the treatment pattern up to the most recent visit prior to t, $g({\bar{a}}_{\left\lfloor t\right\rfloor };\beta_{A})$ is a function of ${\bar{a}}_{\left\lfloor t\right\rfloor }$ to be specified, and $\beta_{A}$ is a vector of log hazard ratios. However, the hazard could take various other forms and we will also consider MSMs based on Aalen's additive hazard model,^45^, ^46^ which have the form

(5) $$h_{T^{{\underline{a}}_{0}}}(t)=\alpha_{0}(t)+g({\bar{a}}_{\left\lfloor t\right\rfloor };\alpha_{A}(t)),$$

where $\alpha_{0}(t)$ is the baseline counterfactual hazard and $\alpha_{A}(t)$ is a vector of time‐dependent coefficients. In both hazard models the g(·) function is chosen to equal zero when ${\bar{a}}_{\left\lfloor t\right\rfloor }=0$. In (3) we also showed that the causal estimand can be expressed in terms of a conditional hazard. For this the MSM for the conditional hazard includes conditioning on (some components of) the covariate history at time t=0, which we denote V. Proportional hazards and additive hazards forms for the conditional hazard are

(6) $$h_{T^{{\underline{a}}_{0}}}(t|V)=h_{0}(t)e^{g({\bar{a}}_{\left\lfloor t\right\rfloor };\beta_{A})+\beta_{L}^{⊤}V},$$

and

(7) $$h_{T^{{\underline{a}}_{0}}}(t|V)=\alpha_{0}(t)+g({\bar{a}}_{\left\lfloor t\right\rfloor };\alpha_{A}(t))+\alpha_{L}^{⊤}(t)V.$$

The hazard models in (4) to (7) are generic forms for the MSM. They could be extended to include interactions between ${\bar{a}}_{\left\lfloor t\right\rfloor }$ and V. Below we consider specific forms for the MSM that are used in the MSM‐IPTW and sequential trials approaches, and we specify V in terms of Z and L. Both methods involve specification of an MSM for the hazard $h_{T^{{\underline{a}}_{0}}}(t)$ or the conditional hazard $h_{T^{{\underline{a}}_{0}}}(t|V)$, followed by estimation using observational data under the assumptions stated above.

For the Cox MSM in (4) the survival probabilities required for the estimand in (1) can be written

(8) $$\begin{array}{ll} \mathrm{Pr}(T^{{\underline{a}}_{0}}>\tau ) & =\exp\left\{-e^{g(a_{0};\beta_{A})}\int_{0}^{1}h_{0}(v)dv-e^{g({\bar{a}}_{1};\beta_{A})}\int_{1}^{2}h_{0}(v)dv\cdots -e^{g({\bar{a}}_{\left\lfloor \tau \right\rfloor };\beta_{A})}\int_{\left\lfloor \tau \right\rfloor }^{\tau }h_{0}(v)dv\right\}. \end{array}$$

For the Aalen MSM in (5) we have

(9) $$\mathrm{Pr}(T^{{\underline{a}}_{0}}>\tau )=\exp\left\{-\int_{0}^{\tau }\alpha_{0}(v)dv-\int_{0}^{1}g(a_{0};\alpha_{A}(v))dv-\int_{1}^{2}g({\bar{a}}_{1};\alpha_{A}(v))dv\cdots -\int_{\left\lfloor \tau \right\rfloor }^{\tau }g({\bar{a}}_{\left\lfloor \tau \right\rfloor };\alpha_{A}(v))dv\right\}.$$

Similar expressions can be used to write the conditional probabilities $\mathrm{Pr}(T^{{\underline{a}}_{0}=a}>\tau |V)$ in terms of the conditional MSM in (6) or (7), which can then be used to estimate the marginal probabilities $\mathrm{Pr}(T^{{\underline{a}}_{0}=a}>\tau )$ using (3). Further details on estimation are discussed below.

### 4.2 Estimation using MSM‐IPTW

#### 4.2.1 Specifying and estimating the MSM

In the MSM‐IPTW approach, the data used for the analysis includes individuals followed‐up from the first time at which they met the trial emulation eligibility criteria, denoted k=0 (see Table 1). This is the time at which the decision to initiate the “always treated” or “never treated” strategy could have been made, and t denotes follow‐up time (ie, t corresponds to k). Individuals are assigned time‐dependent weights to address time‐dependent confounding and the MSM is fitted to the observed weighted data. Consider first the MSM that does not condition on any covariate history at time t=0. In a simple version of the MSM, the hazard at time t is assumed to depend only on the current level of treatment: $g({\bar{a}}_{\left\lfloor t\right\rfloor };\beta_{A})=\beta_{A}a_{\left\lfloor t\right\rfloor }$ (model (4)) or $g({\bar{a}}_{\left\lfloor t\right\rfloor };\alpha_{A}(t))=\alpha_{A}(t)a_{\left\lfloor t\right\rfloor }$ (model (5)). Other options include that the hazard depends on duration of treatment, $g({\bar{a}}_{\left\lfloor t\right\rfloor };\beta_{A})=\beta_{A}\sum_{k=0}^{\left\lfloor t\right\rfloor }a_{k}$ or $g({\bar{a}}_{\left\lfloor t\right\rfloor };\alpha_{A}(t))=\alpha_{A}(t)\sum_{k=0}^{\left\lfloor t\right\rfloor }a_{k}$; or on the history of treatment in a more general way, $g({\bar{a}}_{\left\lfloor t\right\rfloor };\beta_{A})=\sum_{k=0}^{\left\lfloor t\right\rfloor }\beta_{Ak}a_{k}$ or $g({\bar{a}}_{\left\lfloor t\right\rfloor };\alpha_{A}(t))=\sum_{k=0}^{\left\lfloor t\right\rfloor }\alpha_{Ak}(t)a_{k}$. The choice of the way in which the hazard depends on the treatment history could be informed by subject matter knowledge. In practice, however, it may be difficult to elicit this information and the choice could depend to some extent on the sample size, with larger sample sizes permitting more flexible forms for the hazard without too much loss of precision. The weight at time t for a given individual is the inverse of the product of conditional probabilities of them having had their observed treatment pattern up to time t given their past treatment status and time‐dependent covariate history, ${\bar{L}}_{\left\lfloor t\right\rfloor },Z$. These weights can be large for some individuals, which induces large uncertainty in estimates from the MSM, and stabilized weights are typically used instead. Details on the weights are provided in the Supplementary Material (Section 2).

Next we consider the MSM that conditions on the covariate history at time t=0. In this case V in Equations (6) and (9) is ${\bar{L}}_{0},Z$. In this case we also need to correctly specify how ${\bar{L}}_{0},Z$ impact on the hazard, including consideration of any interaction terms between ${\bar{a}}_{\left\lfloor t\right\rfloor }$ and ${\bar{L}}_{0},Z$. Inclusion of interactions with ${\bar{L}}_{0},Z$ may also be informed by subject matter knowledge and could in theory be assessed using statistical tests, which should be based on an appropriate method for estimating standard errors such as bootstrapping (see below). If stabilized weights are estimated by including the covariate history at time t=0, that is, ${\bar{L}}_{0},Z$, in the model in the numerator of the stabilized weights then ${\bar{L}}_{0},Z$ must also be included in the MSM, that is, the MSM should be for a conditional hazard $h_{T^{{\underline{a}}_{0}}}(t|{\bar{L}}_{0},Z)$. An MSM based on a conditional hazard can also be used in combination with unstabilized or partially stabilized weights. In that case, however, the conditioning variables are not being used to control confounding, as that is achieved via the weights.

In the data there is likely to be censoring of individuals due to loss‐to‐follow‐up or for other reasons. Censoring that depends on time‐updated covariates, or on baseline covariates that are not conditioned on in the MSM (ie, not included in the set of baseline adjustment variables in the MSM, if any), is informative, and this should be accounted for in the analysis to avoid bias. Inverse probability of censoring weights (IPCW) can be used to address this. The total weight at a given time is the product of the IPTW and IPCW at that time.

#### 4.2.2 Estimating the causal estimand

Having fitted a MSM using a Cox model for the marginal hazard as in (4), we use the expression in (8) to obtain estimates of the marginal probabilities of interest. In (8) the integrated (cumulative) baseline hazards can be estimated using an IPTW Breslow's estimator (see Section 2 in the Supplementary Material). Alternatively, having fitted a MSM using an Aalen additive model for the marginal hazard (5), we use the expression in (9), where the integrals are estimated as the cumulative coefficients estimated in the Aalen additive hazard model fitting process. When using an MSM for a marginal hazard the resulting marginal survival probabilities refer to the population at time zero. If using an MSM with conditioning on ${\bar{L}}_{0},Z$ we can use the fitted MSM to estimate conditional survival probabilities $\mathrm{Pr}(T^{{\underline{a}}_{0}=a}>\tau |{\bar{L}}_{0},Z)$. These conditional estimates can then be used to obtain estimates of marginal probabilities $\mathrm{Pr}(T^{{\underline{a}}_{0}=a}>\tau )$ for a population of interest using empirical standardization. Here we have a choice about the population to which the marginal probabilities refer, in terms of their distribution of ${\bar{L}}_{0},Z$. In the motivating example, we let 𝒞 denote the set of individuals meeting the trial emulation eligibility criteria in 2018, and the number of individuals in 𝒞 is denoted $n_{𝒞}$. We let ${\bar{l}}_{i},z_{i}$ denote the observed values of the covariate history for individual i∈𝒞 in 2018. The empirical standardization formula is

(10) $$\begin{array}{ll} \hat{\mathrm{Pr}}(T^{{\underline{a}}_{0}=a}>\tau ) & =\frac{1}{n_{𝒞}}\sum_{i\in 𝒞}\hat{\mathrm{Pr}}(T_{{\underline{a}}_{0}=a}>\tau |{\bar{L}}_{0}={\bar{l}}_{i},Z=z_{i})=\frac{1}{n_{𝒞}}\sum_{i\in 𝒞}\exp\left\{-{\hat{H}}_{T^{{\underline{a}}_{0}=a}}(\tau |{\bar{L}}_{0}={\bar{l}}_{i},Z=z_{i})\right\}. \end{array}$$

where ${\hat{H}}_{T^{{\underline{a}}_{0}=a}}(\tau |{\bar{L}}_{0}={\bar{l}}_{i},Z=z_{i})$ is the estimate of the conditional cumulative hazard at time τ. In practice, the empirical standardization can be done by creating two copies of each individual in 𝒞 (sometimes called “cloning”) and setting $A_{0}=A_{1}=\ldots =A_{\left\lfloor \tau \right\rfloor }=1$ for one copy and $A_{0}=A_{1}=\ldots =A_{\left\lfloor \tau \right\rfloor }=0$ for the other copy. The covariate values ${\bar{l}}_{i},z_{i}$ are the same for both copies. The estimated conditional survival probabilities are then obtained for each individual under both treatment regimes (ie, for both copies), and then the average calculated across individuals under both treatment regimes. A similar empirical or ‘plug‐in’ approach for estimating risks based on MSMs was described by Young et al.^27^ See also Chen and Tsiatis^47^ and Daniel et al.^48^

If it is of interest to estimate the causal risk difference in (1) for several time horizons τ, it is recommended to fit the MSM using all event times up to $\tau_{\max}$ to obtain risk difference estimates for all horizons $\tau \leq \tau_{\max}$, rather than fitting separate MSMs for different time horizons. This is to guarantee that estimated survival curves are monotone.

Confidence intervals for the estimated causal risk difference can be obtained by bootstrapping. The weights models and the MSM are fitted in each bootstrap sample, so that the bootstrap confidence intervals capture the uncertainty in the estimation of the weights as well as in the MSM.

### 4.3 Estimation using the sequential trials approach

#### 4.3.1 Creation of trials and use of MSMs

  The sequential trials approach takes advantage of the fact that individuals can meet the trial emulation eligibility criteria at more than one visit during their follow‐up, and therefore have data consistent with initiating the “always treated” or “never treated” strategy at more than one visit k (Table 1). This is the key difference between the MSM‐IPTW approach and the sequential trials approach. Another important difference is that the sequential trials approach focuses only on two sustained treatment regimes of interest—“always treated” and “never treated”—whereas the MSM‐IPTW approach permits estimation of risks under any longitudinal treatment regime. The two methods are compared in detail in Section 5. Here we focus on describing the sequential trials approach.

In the sequential trials approach the trial emulation eligibility criteria are applied at each visit k=0,1,… to form a sequence of “trials.” An individual can contribute to a “trial” starting from any visit k=0,1,2,… at which they meet the criteria. The time t=0 in a given trial denotes the time since the start of the trial. In typical applications of the sequential trials approach, individuals who have previously used the treatment are not eligible for inclusion in a given trial, and so all individuals included in the kth trial have ${\bar{A}}_{k-1}=0$. Those included in the kth trial therefore include “initiators” who start treatment at visit k ($A_{k}=1$) and “non‐initiators” who remain untreated at visit k ($A_{k}=0$). Noninitiators in trial k can appear as initiators in a later trial (k+1,…). Individuals can appear as initiators in only one trial, but as noninitiators in several trials. In our motivating example we relax this slightly and only require individuals not to have been treated for three prior years in order to be included in a given trial according to the trial emulation eligibility criteria, that is, a person is eligible at visit k if $A_{k-1}=A_{k-2}=A_{k-3}=0$. In the example, therefore, an individual could appear as a new initiator in more than one trial, and also as a new initiator in one trial and a noninitiator in a trial more than 3 years later. To simplify the notation below we assume that individuals meeting the trial emulation eligibility criteria for the kth trial have ${\bar{A}}_{k-1}=0$, but the details are unchanged in the slightly relaxed situation of the motivating example.

Previous descriptions of the sequential trials approach have described the analysis models used but have not expressed these in terms of MSMs using the counterfactual framework.^4^, ^5^ Here we consider MSMs for use in the sequential trials approach, focusing on MSMs that condition on the covariate history at the start of the trial, $h_{T^{{\underline{a}}_{0}}}(t|V)$, where V corresponds to ${\bar{L}}_{k},Z$ for trial k. First consider the trial starting at visit k=0. Let $h_{0,T^{{\underline{a}}_{0}=a}}(t|{\bar{L}}_{0},Z)$ denote the hazard at time t after visit 0 under the possibly counter‐to‐fact regime of being “always treated” (a=1) or “never treated” (a=0) from visit 0 onwards, conditional on ${\bar{L}}_{0},Z$. MSMs for this hazard using a Cox model and Aalen's additive hazard model are

(11) $$h_{0,T^{{\underline{a}}_{0}=a}}(t|{\bar{L}}_{0},Z)=h_{00}(t)\exp\left\{f(t;\beta_{A0})a+\beta_{L0}^{⊤}{\bar{L}}_{0}+\beta_{Z0}^{⊤}Z\right\},$$

and

(12) $$h_{0,T^{{\underline{a}}_{0}=a}}(t|{\bar{L}}_{0},Z)=\alpha_{00}(t)+\alpha_{A0}(t)a+\alpha_{L0}^{⊤}(t){\bar{L}}_{0}+\alpha_{Z0}^{⊤}(t)Z.$$

We extend this to a trial starting at any visit k and let $h_{k,T^{{\underline{a}}_{0}=a}}(t|{\bar{A}}_{k-1}=0,{\bar{L}}_{k},Z)$ denote the hazard at time t after visit k under this possibly counter‐to‐fact treatment regime, conditional on the time‐fixed covariates Z and the characteristics ${\bar{L}}_{k}$ at the start of trial k. We note that the subscript 0 on ${\underline{a}}_{0}$ in the counterfactual event time $T_{{\underline{a}}_{0}}$ refers to time t=0 (the start of a given trial) rather than to visit k=0. MSMs for this hazard using a Cox model and Aalen's additive hazard model are therefore

(13) $$h_{k,T^{{\underline{a}}_{0}=a}}(t|{\bar{A}}_{k-1}=0,{\bar{L}}_{k},Z)=h_{0k}(t)\exp\left\{f(t;\beta_{Ak})a+\beta_{Lk}^{⊤}{\bar{L}}_{k}+\beta_{Zk}^{⊤}Z\right\},$$

and

(14) $$h_{k,T^{{\underline{a}}_{0}=a}}(t|{\bar{A}}_{k-1}=0,{\bar{L}}_{k},Z)=\alpha_{0k}(t)+\alpha_{Ak}(t)a+\alpha_{Lk}^{⊤}(t){\bar{L}}_{k}+\alpha_{Z}^{⊤}(t)Z.$$

The conditioning on ${\bar{L}}_{k},Z$ is included to control for confounding by covariates at the start of the trial. Such confounding could alternatively be controlled using weights, but conditioning on covariate history at the start of the trial is a feature of the previously‐described sequential trials analysis methods, which we discuss further below. In the Cox MSM (13) the hazards in the treated and untreated could be assumed proportional, $f(t;\beta_{Ak})=\beta_{Ak}$, or we could allow the hazard to depend on duration of treatment, for example, $f(t;\beta_{Ak})=\beta_{Ak,0}+\beta_{Ak,1}t$. The additive hazards MSM in (14) naturally incorporates a flexible time‐dependent effect of treatment on the hazard. Both models could be extended to include interactions between a and ${\bar{L}}_{k},Z$.

#### 4.3.2 Estimation using inverse probability of artificial‐censoring weights

The MSMs in (13) and (14) cannot be estimated directly from the observational data because not all individuals meeting the trial emulation eligibility criteria at visit k are then “always treated” (${\underline{A}}_{k}=1$) or “never treated” (${\underline{A}}_{k}=0$). In the approach as described by Hernan et al^4^ (their “adherence‐adjusted” approach) and Gran et al^5^ individuals are artificially censored at the visit at which they switch from their treatment group at the start of the trial. That is, in the trial starting at visit k, individuals are censored at the earliest visit m (m>k) such that $A_{m}\neq A_{k}$. We refer to this as *artificial censoring*. It results in a modified dataset in which, in trial k, all individuals have either ${\underline{A}}_{k}=0$ or ${\underline{A}}_{k}=1$ up to the earliest of their event time, their actual censoring time, or their artificial censoring time. Treatment switching is expected to depend on time‐dependent covariates that are also associated with the outcome, as illustrated in the DAG in Figure 1, meaning that the artificial censoring is informative. This is addressed using weights, which we refer to as inverse probability of artificial‐censoring weights (IPACW). The IPACW at times 0<t<1 (in each trial k) is 1. The IPACW at times l≤t<l+1 (l≥1) is the inverse of the product of conditional probabilities that the individual's treatment status up to time l remained the same as their treatment status at the start of the trial (time t=0), conditional on their observed covariates up to time l. After estimating the IPACW, the MSMs in (13) and (14) can then be fitted using weighted regression using the time‐dependent IPACW, with a in the hazard model replaced by the observed treatment status at the start of the trial, that is, $A_{k}$ in trial k, noting that treatment status is forced to be the same at all times t due to the artificial censoring. The conditioning on ${\bar{L}}_{k},Z$ controls for confounding of the association between treatment initiation at time 0 and the hazard. Estimating the MSMs in this way is valid under the same assumptions of no interference, positivity, consistency and conditional exchangeability, as are required for the MSM‐IPTW analysis. Further details on the IPACW are provided in the Supplementary Material (Section 2). Hernan et al^4^ used logistic regression to estimate the IPACW, whereas Gran et al^5^ used Aalen's additive hazard model.

#### 4.3.3 A combined analysis across trials

The MSMs in (13) and (14) could be fitted separately in each trial starting at times k=0,1,…. However, the strength of the sequential trials approach comes from the potential for a combined analysis across trials. Some or all parameters of the MSMs in (13) and (14) could be assumed common across trials (ie, the same for all k). The common parameters can then be estimated by fitting the hazard models using the observed data combined across trials. When the MSMs are fitted combined across trials, the MSM for each trial k preferably conditions on the same amount of history of $L_{k}$. For example, we may wish to allow the hazard for each trial k to depend on $L_{k}$ and $L_{k-1}$. The amount of history to condition on should be based on what is considered to be needed to control for confounding, which can be encompassed in a DAG. We let ${\bar{L}}_{k-k^{*},k}=\{L_{k-k^{*}},\ldots ,L_{k}\}$ denote the extent of the history of ${\bar{L}}_{k}$ that we wish to condition on in the MSM. If we allowed the MSMs for the conditional hazards for different trials to depend on different amounts of history of ${\bar{L}}_{k}$ then justification for a combined model may be challenging.

It may be reasonable in many settings to assume that the impact of the treatment on the hazard at a given time post‐initiation of the treatment does not depend on the visit k at which treatment was initiated, that is, $\beta_{Ak}=\beta_{A}$ in model (13) and $\alpha_{Ak}(t)=\alpha_{A}(t)$ in model (14) for all k. Similarly the coefficients for ${\bar{L}}_{k-k^{*},k}$ and Z could be assumed constant across trials. Often the observation time scale k will be in a sense arbitrary. In our motivating example we define time k=0 as the first time during the period 2008 to 2017 that an individual is observed in the UK CF Registry and first meets the trial emulation eligibility criteria. For many individuals this is 2008 (see Section 7). Provided any elements of time (eg, age, calendar year) that affect the hazard are included in the set of covariates ${\bar{L}}_{k-k^{*},k}$, the baseline hazard may also be assumed common across trials, that is, $h_{0k}(t)=h_{0}(t)$ or $\alpha_{0k}(t)=\alpha_{0}(t)$. The models used to obtain the IPACW could be fitted separately in each trial or combined across trials.

As in the MSM‐IPTW approach, censoring of individuals due to loss‐to‐follow‐up or for other reasons, and which depends on time‐updated covariates, can be handled using IPCW. In the sequential trials analysis these weights are distinct from the IPACW used to address the artificial censoring, and the total weight at a given time is given by the product of these two types of weight. Gran et al^5^ did not distinguish between artificial censoring in data set‐up for the sequential trials approach and censoring due to drop‐out in their analysis, and used a single weight to account for both of these.

#### 4.3.4 Estimating the causal estimand

After estimating the MSM using the sequential trials data the expression in (3) can be used to obtain estimates of conditional survival probabilities $\mathrm{Pr}(T^{{\underline{a}}_{0}=a}>\tau |{\bar{A}}_{k‐1}=0,{\bar{L}}_{k-k^{*},k},Z)$. These conditional probabilities can then be used to obtain the marginal probabilities used in the causal estimand in (1). As in the MSM‐IPTW approach when using an MSM that is conditional on ${\bar{L}}_{0},Z$, we have some choice here about the population for which the marginal probabilities are obtained. Suppose we decided to fit a Cox MSM of the form

(15) $$h_{k,T^{{\underline{a}}_{0}=a}}(t|{\bar{A}}_{k-1}=0,{\bar{L}}_{k-k^{*},k},Z)=h_{0}(t)\exp\left\{\beta_{A}a+\beta_{L}^{⊤}{\bar{L}}_{k-k^{*},k}+\beta_{Z}^{⊤}Z\right\},$$

and that this is fitted combined across all trials, therefore giving combined estimates of the model parameters and the baseline hazard. As in Section 4.2 we let 𝒞 denote the observed sample from the population of interest, that is, individuals meeting the trial emulation criteria in 2018 in our motivating example. We let ${\bar{l}}_{i},z_{i}$ denote the observed values of time‐dependent covariates ${\bar{L}}_{k-k^{*},k},Z$ for individual i∈𝒞. Using the Cox MSM in (15) the empirical standardization formula is

(16)

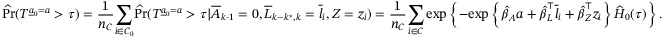


where ${\hat{H}}_{0}(\tau )$ is the estimated cumulative baseline hazard, which is obtained using an IPACW Breslow's estimator (see Section 2 in Data S1). A similar expression can be written if the model in (15) is replaced by an additive hazards model. The standardized probability estimate in (16) targets the same probability as the corresponding expression for the MSM‐IPTW approach in (10). They both refer to a population 𝒞. Both also involve a baseline hazard. The baseline hazards are themselves marginal with respect to covariates not included in the MSM for the conditional hazards, and will refer to different populations in the MSM‐IPTW and sequential trials approaches. However, provided that important aspects of “time” such as age and calendar time are included in the covariate set, we argue that these differences are likely to have negligible impact on estimates. In (15) the baseline hazard is assumed the same across trials. We could alternatively allow the baseline hazard to differ across trials, while assuming that the model coefficients are the same across trials. For this the MSM would be fitted using data combined across trials, but allowing the baseline hazard to differ by trial (ie, using a stratified baseline hazard). When allowing different baseline hazards across trials, care should be taken over which baseline hazard is then used to obtain the marginal risk estimates. In this paper we focus on a sequential trials approach in which the length of possible follow‐up decreases for trials starting at later visits. The baseline hazard corresponding to the trial starting at k=0 may be used to obtain the marginal risk difference estimates for all time horizons $\tau \leq \tau_{\max}$. The baseline hazard from the trial starting at k=1 could only be used to estimate marginal risk difference estimates for time horizons $\tau \leq \tau_{\max}-1$. An alternative would be to use a sequence of trials that all have equal length of follow‐up. In our motivating example, which uses data from 2008 to 2018, there are a maximum of 11 years of follow‐up (from 2008). If we did not use a combined analysis across trials, the parameters in (16) would be trial dependent, and one would then have to consider which set of parameters to use to obtain the marginal risk estimates.

In their descriptions of the sequential trials approach Gran et al (2010)^5^ used the Cox proportional hazards model, and Hernan et al (2008)^4^ used pooled logistic regression across visits, which is equivalent to a Cox regression as the times between visits gets smaller.^49^ Røysland et al^50^ used an additive hazards model, though their aim was to estimate direct and indirect effects, which differs from our aims. Gran et al (2010)^5^ assumed the hazard ratios for treatment in the Cox model to be common across the trials, but allowed a different baseline hazard in each trial. Hernan et al (2008)^4^ allowed the odds ratio for treatment to differ across trials and also performed a test for heterogeneity, though it is unclear whether they allowed separate intercept parameters (analogous to our baseline hazards) across trials. If the coefficient for treatment is assumed the same across trials, it is recommended to assess this assumption using a formal test.

As in the MSM‐IPTW approach, confidence intervals for the estimated causal risk difference can be obtained by bootstrapping. The formation of the sequential trials, estimation of the weights, fitting of the conditional MSMs, and obtaining the risk difference are all repeated in each bootstrap sample.

## 5 COMPARING MSM‐IPTW AND THE SEQUENTIAL TRIALS APPROACH

### 5.1 An illustrative example using a causal tree diagram

In this section we show how the MSM‐IPTW and sequential trials approaches can estimate the same causal estimand using a simple example and non‐parametric estimates. Our aim is to provide insight into how the two approaches use the data differently to estimate the same quantity.

**FIGURE 2:**
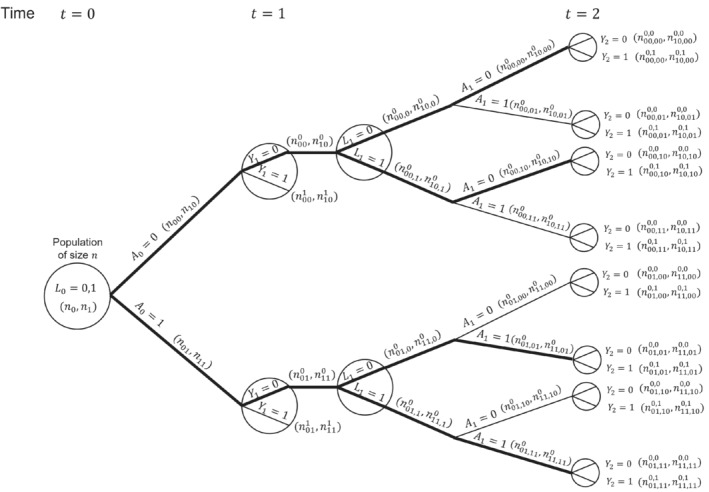
Causal tree diagram illustrating a study with a binary treatment $A_{t}$ and binary covariate $L_{t}$, both measured at two time points (t=0,1). $Y_{t}$ is a discrete time survival outcome. Thick lines indicate branches for groups of individuals who were untreated or treated at both time points.

Consider a situation as depicted in the DAG in Figure 1, but with treatment and covariate information only collected at up to two visits ($L_{0},A_{0}$ measured at time 0, and $L_{1},A_{1}$ measured at time 1) and survival status observed at times 1 ($Y_{1}=I(0<T\leq 1)$) and 2 ($Y_{2}=I(0<T\leq 2)$). We focus on a single binary time‐dependent confounder L and omit the unobserved variable U and any time‐fixed covariates Z. Figure 2 shows a causal tree graph (see for example Richardson and Rotnitzky (2014)^51^), representing the different possible combinations of the variables $L_{0},A_{0},Y_{1}$ up to time 1, and then the different combinations of $L_{1},A_{1},Y_{2}$ among individuals who survive to time 1 ($Y_{1}=0$). A total of n individuals are observed at time 0. The numbers in brackets on the branches of the tree are the number of individuals who followed a given pathway up to that branch. The notation is as follows: $n_{l_{0}}$ denotes the number with $L_{0}=l_{0}$; $n_{l_{0}a_{0}}$ the number with $L_{0}=l_{0},A_{0}=a_{0}$; $n_{l_{0}a_{0}}^{y_{1}}$ the number with $L_{0}=l_{0},A_{0}=a_{0},Y_{1}=y_{1}$; $n_{l_{0}a_{0},l_{1}}^{0}$ the number with $L_{0}=l_{0},A_{0}=a_{0},Y_{1}=0,L_{1}=l_{1}$; $n_{l_{0}a_{0},l_{1}a_{1}}^{0}$ the number with $L_{0}=l_{0},A_{0}=a_{0},Y_{1}=0,L_{1}=l_{1},A_{1}=a_{1}$; and $n_{l_{0}a_{0},l_{1}a_{1}}^{0,y_{2}}$ the number with $L_{0}=l_{0},A_{0}=a_{0},Y_{1}=0,L_{1}=l_{1},A_{1}=a_{1},Y_{2}=y_{2}$.

**FIGURE 3:**
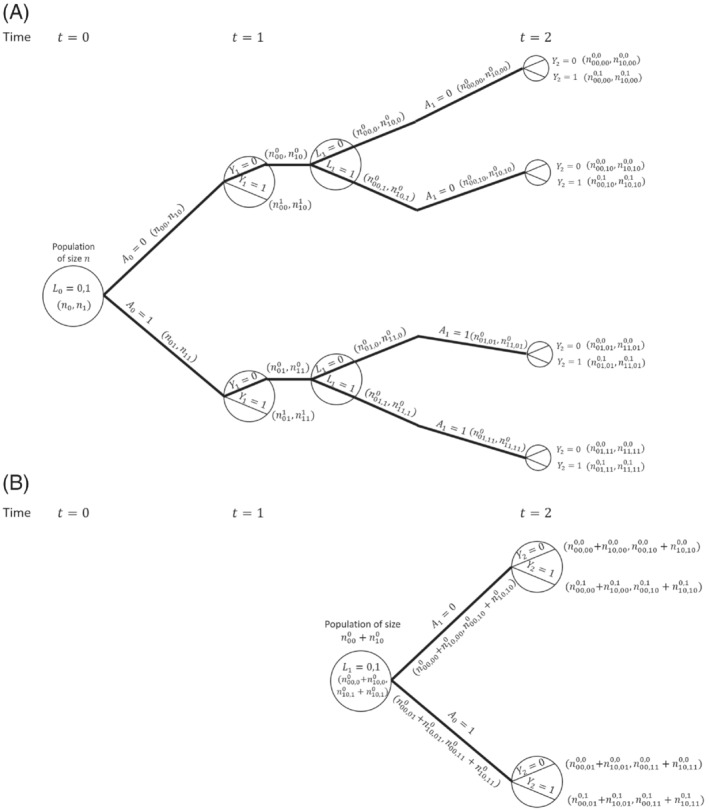
Illustration of the sequential trials approach using causal tree diagram from Figure 2. (A) Trial starting at t=0 . (B) Trial starting at t=1. (A) In the trial starting at time t=0 individuals with $Y_{1}=0$ are censored at time 1 if $A_{1}\neq A_{0}$. (B) The trial starting at time t=1 is restricted to individuals with $A_{0}=0$ (and $Y_{1}=0$).

Our interest is in comparing survival probabilities under the two treatment regimes of “always treated” and “never treated.” The branches representing these two treatment regimes starting from k=0 are shown with thick lines in the causal tree diagram. The MSM‐IPTW analysis makes use of the causal tree diagram as depicted in Figure 2. Under the sequential trials approach, we create a trial starting at time 0 and a trial starting at time 1. In the trial starting at time 0 individuals who survive to time 1 are censored at time 1 if $A_{1}\neq A_{0}$, that is, not all branches after time 1 are used. The trial starting at time 1 is restricted to individuals with $A_{0}=0$ and $Y_{1}=0$. The sections of the causal tree diagram used for these two trials are shown in Figure 3.

Consider the probability of survival to time 1 with treatment a, $\mathrm{Pr}(Y_{1}^{a_{0}=a}=0)$. The MSM‐IPTW approach estimates this using the result that (under the conditions of consistency, positivity and conditional exchangeability)

(17) $$\mathrm{Pr}({Y_{1}}^{a_{0}=a}=0)=E\left\{\frac{I(A_{0}=a)(1-Y_{1})}{\mathrm{Pr}(A_{0}=a|L_{0})}\right\}.$$

Based on the tree diagram, a nonparametric estimate of this is

(18) $$\hat{\mathrm{Pr}}(Y_{1}^{a_{0}=a}=0)=\frac{1}{n}\left\{n_{0a}^{0}{\left(\frac{n_{0a}}{n_{0}}\right)}^{-1}+n_{1a}^{0}{\left(\frac{n_{1a}}{n_{1}}\right)}^{-1}\right\},$$

where the two contributions within the outer brackets come from individuals with $L_{0}=0$ and $L_{0}=1$. On the other hand, the sequential trial starting at time 0 estimates $\mathrm{Pr}(Y_{1}^{a_{0}=a}=0|L_{0})$. By consistency and conditional exchangeability we have $\mathrm{Pr}(Y_{1}^{a_{0}=a}=0|L_{0})=\mathrm{Pr}(Y_{1}=0|A_{0}=a,L_{0})$. Based on the tree diagram, a non‐parametric estimate of this using the trial starting at time 0 is $\mathrm{Pr}(Y_{1}=0|A_{0}=a,L_{0}=l_{0})=\frac{n_{l_{0}a}^{0}}{n_{l_{0}a}},\;l_{0}=0,1$. Using the result that $\mathrm{Pr}(Y_{1}^{a_{0}=a}=0)=\sum_{l_{0}}\mathrm{Pr}(Y_{1}=0|A_{0}=a,L_{0}=l_{0})\mathrm{Pr}(L_{0}=l_{0})$ (assuming consistency and conditional exchangeability) it follows that a nonparametric estimate of the marginal probability $\mathrm{Pr}(Y_{1}^{a_{0}=a}=0)$ based on the trial starting at time 0 is

(19) $$\hat{\mathrm{Pr}}(Y_{1}^{a_{0}=a}=0)=\frac{n_{0a}^{0}}{n_{0a}}\frac{n_{0}}{n}+\frac{n_{1a}^{0}}{n_{1a}}\frac{n_{1}}{n},$$

which is the same as the estimate obtained using MSM‐IPTW (ie, Equation 18). Therefore the MSM‐IPTW and sequential trials approaches (using the trial starting at visit 0) give identical estimates of $\mathrm{Pr}(Y_{1}^{a_{0}=a}=0)$. This equivalence between inverse probability weighted estimates and standardized estimates obtained using the g‐formula in the nonparametric setting is well established (see eg, References 43 and 52). In the sequential trials approach, the trial starting at time 1 (so that t=0 corresponds to k=1) can be used to estimate the probability of survival for 1 year under a given treatment strategy conditional on survival to time 1 and on $A_{0}=0$. By consistency and conditional exchangeability, and assuming that conditioning on $L_{1}$ is sufficient to control for confounding of the association between $A_{1}$ and $Y_{2}$, the nonparametric estimate is

(20) $$\hat{\mathrm{Pr}}(Y_{2}^{a_{0}=a}=0|Y_{1}=0,A_{0}=0)=\left(\frac{n_{00,0a}^{0,0}+n_{10,0a}^{0,0}}{n_{00,0a}^{0}+n_{10,0a}^{0}}\right)\left(\frac{n_{00,0}^{0}+n_{10,0}^{0}}{n_{00}^{0}+n_{10}^{0}}\right)+\left(\frac{n_{00,1a}^{0,0}+n_{10,1a}^{0,0}}{n_{00,1a}^{0}+n_{10,1a}^{0}}\right)\left(\frac{n_{00,1}^{0}+n_{10,1}^{0}}{n_{00}^{0}+n_{10}^{0}}\right),$$

where the two contributions come from individuals with $L_{1}=0$ and $L_{1}=1$. The marginal probability estimate $\hat{\mathrm{Pr}}(Y_{1}^{a_{0}=a}=0)$ refers to a population in which $\mathrm{Pr}(L_{0}=1)=n_{1}/n$, whereas the marginal probability estimate $\hat{\mathrm{Pr}}(Y_{2}^{a_{0}=a}=0|Y_{1}=0,A_{0}=0)$ refers to a population with a different distribution of the covariate measured at the start of the trial, $L_{1}$. The estimate from the trial starting at time 1 could alternatively be standardized to the distribution of $L_{0}$ at time 0:

(21) $${\hat{\mathrm{Pr}}}^{\text{Std}}(Y_{2}^{a_{0}=a}=0|Y_{1}=0,A_{0}=0)=\left(\frac{n_{00,0a}^{0,0}+n_{10,0a}^{0,0}}{n_{00,0a}^{0}+n_{10,0a}^{0}}\right)\left(\frac{n_{0}}{n}\right)+\left(\frac{n_{00,1a}^{0,0}+n_{10,1a}^{0,0}}{n_{00,1a}^{0}+n_{10,1a}^{0}}\right)\left(\frac{n_{1}}{n}\right).$$

Under the assumption that ${\mathrm{Pr}}^{\text{Std}}(Y_{2}^{a_{0}=a}=0|Y_{1}=0,A_{0}=0)=\mathrm{Pr}(Y_{1}^{a_{0}=a}=0)$, a combined estimate of $\mathrm{Pr}(Y_{1}^{a_{0}=a}=0)$ could be obtained from the two trials, for example, using an inverse‐variance‐weighted combination of the estimates from the two trials (Equations 19 and 21).

Considering again the trial starting at time 0, we can also show that non‐parametric estimates of $\mathrm{Pr}(Y_{2}^{{\underline{a}}_{0}=a}=0)$ from the two methods are the same. The MSM‐IPTW approach uses

(22) $$\mathrm{Pr}(Y_{2}^{{\underline{a}}_{0}=a}=0)=E\left\{\frac{I(A_{0}=a,A_{1}=a)(1-Y_{1})(1-Y_{2})}{\mathrm{Pr}(A_{0}=a|L_{0})\mathrm{Pr}(A_{1}=a|Y_{1}=0,A_{0}=a,L_{0},L_{1})}\right\}.$$

Based on the tree diagram, a nonparametric estimate of this is

(23) $$\begin{array}{llll} \hat{\mathrm{Pr}}(Y_{2}^{{\underline{a}}_{0}=a}=0)= & \frac{1}{n}\left\{n_{0a,0a}^{0,0}{\left(\frac{n_{0a}}{n_{0}}\right)}^{-1}{\left(\frac{n_{0a,0a}^{0}}{n_{0a,0}^{0}}\right)}^{-1}+n_{0a,1a}^{0,0}{\left(\frac{n_{0a}}{n_{0}}\right)}^{-1}{\left(\frac{n_{0a,1a}^{0}}{n_{0a,1}^{0}}\right)}^{-1}\right.\; & & \\ & +\left.n_{1a,0a}^{0,0}{\left(\frac{n_{1a}}{n_{1}}\right)}^{-1}{\left(\frac{n_{1a,0a}^{0}}{n_{1a,0}^{0}}\right)}^{-1}+n_{1a,1a}^{0,0}{\left(\frac{n_{1a}}{n_{1}}\right)}^{-1}{\left(\frac{n_{1a,1a}^{0}}{n_{1a,1}^{0}}\right)}^{-1}\right\},\; & & \end{array}$$

where the four contributions within the outer brackets come from individuals with the four possible combinations of $\left(L_{0},L_{1}\right)$.

The sequential trials approach estimates $\mathrm{Pr}(Y_{2}^{{\underline{a}}_{0}=a}=0|L_{0})$, which can be written

(24) $$\mathrm{Pr}(Y_{2}^{{\underline{a}}_{0}=a}=0|L_{0})=\mathrm{Pr}(Y_{2}^{{\underline{a}}_{0}=a}=0|Y_{1}^{a_{0}=a}=0,L_{0})\mathrm{Pr}(Y_{1}^{a_{0}=a}=0|L_{0}).$$

The term $\mathrm{Pr}(Y_{1}^{a_{0}=a}=0|L_{0})$ was considered above. The first term, $\mathrm{Pr}(Y_{2}^{{\underline{a}}_{0}=a}=0|Y_{1}^{a_{0}=a}=0,L_{0})$, is estimated by IPACW, because individuals with $A_{1}\neq A_{0}$ are censored at time 1, and the remaining individuals with $A_{1}=A_{0}$ are weighted by $\mathrm{Pr}{\left(A_{1}=a|A_{0}=a,L_{0},L_{1}\right)}^{-1}$. Under the assumptions of consistency, positivity and conditional exchangeability we can write

(25) $$\mathrm{Pr}(Y_{2}^{{\underline{a}}_{0}=a}=0|Y_{1}^{a_{0}=a}=0,L_{0})=E\left\{\frac{I(A_{1}=a)(1-Y_{2})}{\mathrm{Pr}(A_{1}=a|Y_{1}=0,A_{0}=a,L_{0},L_{1})}|Y_{1}=0,A_{0}=a,L_{0}\right\}.$$

Based on the tree diagram, a nonparametric estimate of this is

(26) $$\begin{array}{llll} \hat{\mathrm{Pr}}(Y_{2}^{{\underline{a}}_{0}=a}=0|Y_{1}^{a_{0}=a}=0,L_{0}=l_{0}) & =\hat{\mathrm{Pr}}(Y_{2}^{{\underline{a}}_{0}=a}=0|Y_{1}=0,A_{0}=a,L_{0}=l_{0})\; & & \\ & =\frac{1}{n_{l_{0}a}^{0}}\left\{n_{l_{0}a,0a}^{0,0}{\left(\frac{n_{l_{0}a,0a}^{0}}{n_{l_{0}a,0}^{0}}\right)}^{-1}+n_{l_{0}a,1a}^{0,0}{\left(\frac{n_{l_{0}a,1a}^{0}}{n_{l_{0}a,1}^{0}}\right)}^{-1}\right\}.\; & & \end{array}$$

Using this result along with the estimate $\hat{\mathrm{Pr}}(Y_{1}^{{\underline{a}}_{0}=a}=0|L_{0}=l_{0})=\hat{\mathrm{Pr}}(Y_{1}=0|A_{0}=a,L_{0}=l_{0})=n_{l_{0}a}^{0}/n_{l_{0}a}$, it can be shown that the sequential trials estimate of $\mathrm{Pr}(Y_{2}^{{\underline{a}}_{0}=a}=0)=\sum_{l_{0}=0,1}\mathrm{Pr}(Y_{2}^{{\underline{a}}_{0}=a}=0|L_{0}=l_{0})\mathrm{Pr}(L_{0}=l_{0})$ is the same as the MSM‐IPTW estimate in (23). By comparing (23) and (26) we can see clearly the connection between the weights used in the MSM‐IPTW approach and those used in the sequential trials approach (IPACW).

### 5.2 Specification and estimation of MSMs

In practice, model‐based estimates are typically required instead of nonparametric estimates, as we usually have multiple time‐dependent confounders to consider, including continuous variables. In this section we compare in more detail the forms of the MSMs used in the MSM‐IPTW and sequential trials approaches, how they are estimated using inverse probability weights, and the assumptions made.

A key difference in the MSMs used in the two approaches is that in the MSM‐IPTW approach the MSM for the hazard at time t includes the history of treatment up to time t, ${\bar{a}}_{\left\lfloor t\right\rfloor }$, whereas the MSM used in the sequential trials approach involves only the treatment status assigned at the start of the trial, which is equivalent to the treatment at any time after the start of the trial due to the artificial censoring. The MSM‐IPTW approach therefore requires that the relation between treatment history ${\bar{a}}_{\left\lfloor t\right\rfloor }$ and the hazard is correctly specified, whereas in the sequential trials approach there are limited options for the form of the MSM because only two treatment regimes are considered, because after the artificial censoring all individuals used in the analysis are either “always treated” or “never treated”. In the MSM‐IPTW approach, when covariates ${\bar{L}}_{0},Z$ are excluded from the MSM, the MSM is a fully marginal model—the possibility of interaction between treatment and these covariates is not ruled out but does not have to be modelled. If the MSM used in the MSM‐IPTW approach includes covariates ${\bar{L}}_{0},Z$ then any interactions that exist between treatment and ${\bar{L}}_{0},Z$ must be included in the MSM and correctly specified. MSMs that condition on the covariate history at time 0 are therefore more susceptible to mis‐specification than MSMs for a marginal hazard.

The sequential trials approach uses an MSM for the hazard that conditions on the covariate history at the start of each trial (${\bar{L}}_{k},Z$ for trial k). This approach therefore relies on correct modelling of the association between outcome and the covariate history in each trial. If there are interactions between treatment and the covariate history at the start of each trial then these must be included in the MSM and correctly specified. When using a combined analysis across trials, each trial should include the same amount of covariate history in the MSM for the conditional hazard, which we denoted in Section 4.3 by ${\bar{L}}_{k-k^{*},k}$. If we adjusted for an increasing length of covariate history in successive trials then the hazard model for each trial would be different. Specifying congenial models across trials for a combined analysis would then be difficult or impossible. If we are willing to make the assumption that the effect of treatment on the hazard is the same in all trials, that is, does not depend on the time of treatment initiation, then the sequential trials approach has information about the effect of treatment initiation from several time origins. Under these assumptions, the re‐use of individuals from several time origins in the sequential trials approach could lead to gains in efficiency in the marginal risk difference estimate at some time horizons τ relative to the MSM‐IPTW approach. On the other hand, in the MSM‐IPTW analysis individuals who switch their treatment status during follow‐up continue to contribute information to the analysis, whereas in the sequential trials analysis individuals cease to contribute information when they deviate from their treatment group as determined at the start of a given trial. This means that in the sequential trials analysis the number of individuals with longer term follow‐up will be smaller than in the MSM‐IPTW analysis. To make use of individuals who switch their treatment status during follow‐up, the MSM‐IPTW approach makes modeling assumptions which borrows information across treatment regimes. The sequential trials approach instead borrows information across populations.

The sequential trials approach could use weighting in the first time period instead of adjustment for covariates measured at the start of the trial, though that is not how the method has been described or used to date. Conditioning on covariates measured at the start of the trial in the sequential trials approach enables use of empirical standardization to obtain risk difference estimates for a population with any distribution of the covariates at time 0, though care should be taken not to extrapolate beyond the observed covariate distribution. We have shown how this enables us to estimate the same causal estimand in the sequential trials approach as in the MSM‐IPTW approach.

To fit the MSMs, both approaches require time‐updated weights, and hence the data should be formatted with multiple rows per individual—one for each visit for MSM‐IPTW, and one for each visit within each trial for the sequential trials approach. In the MSM‐IPTW approach without conditioning on ${\bar{L}}_{0},Z$ the IPTW weights are proportional to the IPACW weights used in the sequential trials approach. Because the MSM‐IPTW approach includes individuals following any treatment regime (not just “always treated” or “never treated”), we may expect to see more extreme weights with increasing follow‐up, compared with the weights used in the sequential trials approach.

Because the two approaches use the data differently and because the MSM‐IPTW approach could make use of more extreme weights, it is of interest to investigate the relative efficiency of the two approaches for estimating the causal estimand.

## 6 A SIMULATION STUDY FOR THE COMPARISON OF MSM‐IPTW AND THE SEQUENTIAL TRIALS APPROACH

### 6.1 Simulation plan

In this section we use a simulation study to evaluate and compare the performance of the MSM‐IPTW approach and the sequential trials approach. It is only appropriate to consider the relative efficiency of the two approaches when they target the same causal estimand, and we focus on the estimation of marginal risks and the marginal risk difference as in (1). In Section 4 we considered MSMs for both methods based on Cox models or on additive hazard models. To enable a fair comparison of the methods, we wish to simulate data in such a way that the MSMs used in the two approaches are correctly specified. To ensure this we simulate the time‐to‐event data under an additive hazards model. In Section 3 of the Supplementary Material we show that one can use an additive hazard model for the MSM used in the sequential trials analysis (Equation 14) (with common parameters assumed across trials) and an additive hazard model for the MSM used in the MSM‐IPTW approach (Equation 5 or 7), and that both MSMs can be correctly specified. However, if we use a Cox model for the MSM for the sequential trials analysis (Equation 13) and a Cox model for the MSM used in the MSM‐IPTW approach (Equation 4 or 6), then both MSMs cannot simultaneously be correctly specified in general. This is because the parameters of additive hazard models are collapsible, whereas hazard ratios in the Cox model are noncollapsible. Martinussen and Vansteelandt^22^ explained the implications of collapsibility for the use of Aalen additive hazards models and Cox models in the context of estimating the causal effect of a point treatment on survival. Keogh et al^28^ extended to a longitudinal setting similar to that considered in this paper.

The simulation study makes use of the data generating simulation algorithm for longitudinal and time‐to‐event data described by Keogh et al.^28^ We follow the general recommendations of Morris et al^53^ on the conduct of simulation studies for evaluating statistical methods. R code for replicating the simulation is provided at https://github.com/ruthkeogh/sequential_trials.

#### 6.1.1 Aim

Our aim is to compare the MSM‐IPTW and sequential trials approaches for estimating the effect of a time‐varying treatment on survival, subject to time‐dependent confounding, from longitudinal observational data. Our focus is on a setting in which both approaches target the same causal estimand (see below) and hence it is relevant to assess their relative efficiency. Both methods are expected to produce approximately unbiased estimates when the modeling assumptions are met. We hypothesize that the sequential trials analysis could be more efficient than the MSM‐IPTW analysis in certain settings, depending on the data generating mechanism and time horizon τ. MSM‐IPTW is inefficient when there are extreme weights. The efficiency of the sequential trials approach is expected to depend on the proportion of individuals always in the treatment group or always in the control group, since when many individuals switch over time this would result in few people contributing as follow‐up time increases, and the potential for large weights.

#### 6.1.2 Data generating mechanisms

Data are generated according to the DAG in Figure 1 for n=5000 individuals. Individuals are observed at up to five visits (k=0,1,2,3,4), with administrative censoring at time 5. We consider three simulation scenarios, starting with a “standard” scenario (scenario 1) and then investigating the performance of the methods when certain aspects of the data generating mechanism are varied. The simulation scenarios are outlined in detail in Table 2. In all scenarios, once an individual starts treatment they remain on treatment, that is, for any k, there are no individuals that go from $A_{k}=1$ to $A_{k+1}=0$. Also in all scenarios there is a single time‐dependent confounder L, the model for $L_{k}$ conditional on $A_{k-1}$, $L_{k-1},k$ and U is the same, and event times are generated according to the same conditional additive hazards model

(27) $$h(k|{\bar{A}}_{\left\lfloor k\right\rfloor },{\bar{L}}_{\left\lfloor k\right\rfloor },U)=\alpha_{0}+\alpha_{A}A_{\left\lfloor k\right\rfloor }+\alpha_{L}L_{\left\lfloor k\right\rfloor }+\alpha_{U}U.$$

In scenarios 1 and 2 the log odds ratio for $L_{k}$ in the logistic model for the probability of $A_{k}=1$ is 0.5, giving “moderate” dependence of treatment initiation on $L_{k}$. In scenario 3, the log odds ratio for $L_{k}$ in the logistic model for the probability of $A_{k}=1$ is increased to 3, meaning that there is strong dependence of treatment initiation on $L_{k}$.

**TABLE 2:** Simulation scenarios: data generating mechanisms.

	Data generating mechanism
General data generating mechanism	U∼N(0,0.1)
	$L_{0}∼N(U,1)$, $L_{k}∼N(\delta_{0}+\delta_{L}L_{k-1}+\delta_{A}A_{k-1}+\delta_{T}k+U,1)$ (k=1,2,3,4)
	$\text{logit}\mathrm{Pr}(A_{0}=1|L_{0})=\gamma_{0}+\gamma_{L}L_{0}$
	$\text{logit}\mathrm{Pr}(A_{k}=1|{\bar{A}}_{k-1},{\bar{L}}_{k})=\gamma_{0}+\gamma_{A}A_{k-1}+\gamma_{L}L_{k}$, (k=1,2,3,4)
	$h(k|{\bar{A}}_{\left\lfloor k\right\rfloor },{\bar{L}}_{\left\lfloor k\right\rfloor })=\alpha_{0}+\alpha_{A}A_{\left\lfloor k\right\rfloor }+\alpha_{L}L_{\left\lfloor k\right\rfloor }+\alpha_{U}U$
Scenario 1	$\delta_{0}=0,\delta_{L}=0.8,\delta_{A}=-1,\delta_{T}=0.1$
	$\gamma_{0}=-1,\gamma_{L}=0.5$
	$\alpha_{0}=0.2,\alpha_{A}=-0.04,\alpha_{L}=0.015,\alpha_{U}=0.015$
Scenario 2	$\delta_{0},\delta_{L},\delta_{A},\delta_{T}$: as in scenario 1
	$\gamma_{0}=-3,\gamma_{L}=0.5$
	$\alpha_{0},\alpha_{A},\alpha_{L},\alpha_{U}$: as in scenario 1
Scenario 3	$\delta_{0},\delta_{L},\delta_{A},\delta_{T}$: as in scenario 1
	$\gamma_{0}=-1,\gamma_{L}=3$
	$\alpha_{0},\alpha_{A},\alpha_{L},\alpha_{U}$: as in scenario 1

In scenario 1 the intercept in the logistic model for the probability of $A_{k}=1$ is such that 25% of individuals have $A_{0}=1$. In scenario 2 the intercept in the logistic model for the probability of $A_{k}=1$ is reduced so that the proportion of individuals initiating treatment at a given visit is lower, with approximately 5% having $A_{0}=1$.

In scenarios 1, 2, and 3, respectively, approximately 57%, 62%, and 56% of individuals have the event of interest during the 5 years of follow‐up. We do not include any censoring apart from administrative censoring, though extensions to include this are straightforward. We generated 1000 simulated data sets.

#### 6.1.3 Estimand

The estimand of interest is the marginal risk difference in (1), standardized to the underlying population at k=0 from which our sample of n individuals arises. We consider time horizons on a continuous scale up to time τ=5.

#### 6.1.4 Methods

The data are analysed using the MSM‐IPTW approach and the sequential trials approach. In the MSM‐IPTW approach we considered MSMs with and without conditioning on $L_{0}$. When the conditional additive hazard model is as in (27), it can be shown^28^ that the correct form for the MSM used in the MSM‐IPTW analysis using the MSM that is not conditional on $L_{0}$ is

(28) $$h_{T^{{\underline{a}}_{0}}}(t)=\alpha_{0}(t)+\overset{\left\lfloor t\right\rfloor }{\underset{j=0}{\sum }}{\tilde{\alpha }}_{Aj}(t)a_{\lfloor t\rfloor -j},$$

and the correct form for the MSM conditional on $L_{0}$ is

(29) $$h_{T^{{\underline{a}}_{0}}}(t|L_{0})=\alpha_{0}(t)+\overset{\left\lfloor t\right\rfloor }{\underset{j=0}{\sum }}{\tilde{\alpha }}_{Aj}(t)a_{\lfloor t\rfloor -j}+\alpha_{L}(t)L_{0}.$$

The correct form for the MSM used in the sequential trials analysis, under our data generating mechanism, is

(30) $$h_{k,T^{{\underline{a}}_{0}=a}}(t|{\bar{L}}_{k})=\alpha_{0}(t)+\alpha_{A}(t)a+\alpha_{L}(t)L_{k},\;k=0,\ldots ,4.$$

The coefficients in the fully conditional hazard model in (27) do not depend on time, and this results in the coefficients in the MSM for the sequential trials analysis being a function of time since the start of the trial, but being the same across trials k=0,…,4.

We use stabilized weights for all analyses. In the MSM‐IPTW approach using the MSM in (28) (without conditioning on $L_{0}$) the stabilized IPTW at time t are

(31) $$\overset{\left\lfloor t\right\rfloor }{\underset{k=0}{\prod }}\frac{\mathrm{Pr}(A_{k}|{\bar{A}}_{k-1})}{\mathrm{Pr}(A_{k}|{\bar{L}}_{k},{\bar{A}}_{k-1})},$$

and in the MSM‐IPTW approach using the MSM in (29) (with conditioning on $L_{0}$) the stabilized IPTW at time t are

(32) $$\overset{\left\lfloor t\right\rfloor }{\underset{k=0}{\prod }}\frac{\mathrm{Pr}(A_{k}|{\bar{A}}_{k-1},L_{0})}{\mathrm{Pr}(A_{k}|{\bar{L}}_{k},{\bar{A}}_{k-1})}.$$

In the sequential trials approach the stabilized IPACW at time t (for t>1) after the start of the trial for the trial starting at visit k are (for t>1)

(33) $$\left\{\begin{array}{cc} 1 & \text{for}\;A_{k}=1,\\ \prod_{j=1}^{\left\lfloor t\right\rfloor }\frac{\mathrm{Pr}(A_{k+j}=0|L_{k},{\bar{A}}_{k+j-1}=0)}{\mathrm{Pr}(A_{k+j}=0|{\bar{L}}_{k+j},{\bar{A}}_{k+j-1}=0)} & \text{for}\;A_{k}=0. \end{array}\right.$$

The IPACW are equal to 1 up to time 1 after the start of each trial.

The weights take into account that individuals do not stop treatment once they start. In MSM‐IPTW we have $\mathrm{Pr}(A_{k}=1|{\bar{A}}_{k-1}=1)=1$, $\mathrm{Pr}(A_{k}=1|{\bar{L}}_{k},{\bar{A}}_{k-2},A_{k-1}=1)=1$ and $\mathrm{Pr}(A_{k}=1|{\bar{A}}_{k-2},A_{k-1}=1,L_{0})=1$. All other probabilities used in the weights were estimated using logistic regression models fitted using all visits combined (k=0,…,4). The probabilities $\mathrm{Pr}(A_{k}|{\bar{A}}_{k-2},A_{k-1}=0)$ were estimated using a logistic regression model for $A_{k}$ with a separate intercept for each k, in the subset of individuals with $A_{k-1}=0$. The probabilities $\mathrm{Pr}(A_{k}|{\bar{A}}_{k-2},A_{k-1}=0,L_{0})$ were estimated using a logistic regression model for $A_{k}$ with $L_{0}$ as a covariate, and allowing both the intercept and the coefficients for $L_{0}$ to differ for each k, fitted using the subset of individuals with $A_{k-1}=0$. The probabilities $\mathrm{Pr}(A_{k}|{\bar{L}}_{k},{\bar{A}}_{k-2},A_{k-1}=0)$ were estimated using a logistic regression model for $A_{k}$ with $L_{k}$ as a covariate in the subset of individuals with $A_{k-1}=0$, which is the correct model under our data generating mechanism. Similar models were used to estimate the weights for the sequential trials analysis, but with $L_{0}$ replaced by $L_{k}$ in trial k for the model used to estimate the probabilities in the numerator of the weights in (33). The models were fitted across all trials combined, which was valid under our data generating mechanism.

**FIGURE 4:**
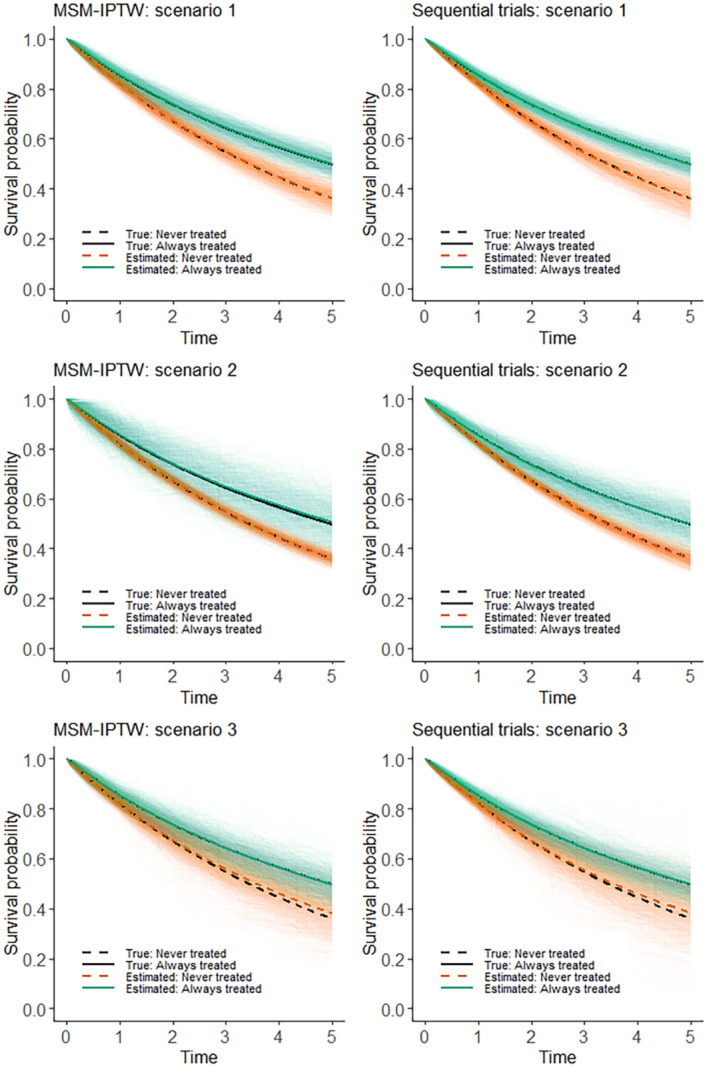
Simulation results: mean estimated survival curves obtained using the sequential trials analysis and the MSM‐IPTW analysis. The faded lines show the estimates from each of the 1000 simulated datasets and the thick lines are the point‐wise averages.

**FIGURE 5:**
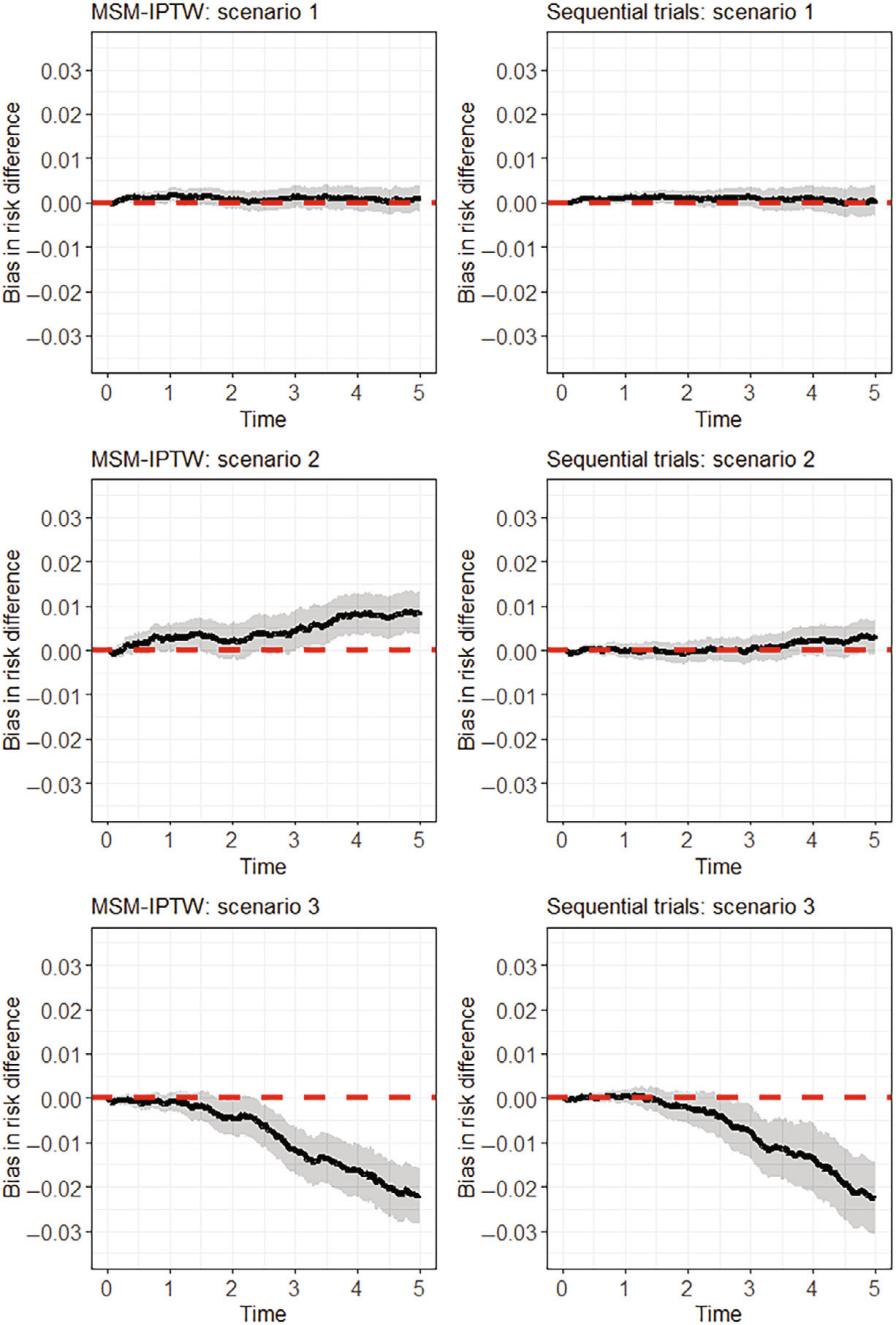
Simulation results: bias in estimation of the risk difference using the sequential trials analysis and the MSM‐IPTW analysis. The black line shows the bias at each time point and the grey area shows the Monte Carlo 95% CI at each time point.

#### 6.1.5 Performance measures

We assess the performance of the methods in terms of bias and efficiency of the risk difference estimates. To obtain the bias we need to know the true values. True values for the survival probabilities and the risk difference were obtained by generating data as though from a large randomized controlled trial, as explained in Keogh et al.^28^ For this, we first generated $L_{0}$ for 1 million individuals, according to the model outlined in Table 1. Two datasets were then created, one in which the 1 million individuals are set to have $A_{k}=1$ (t=0,…,4) (“always treated”) and another in which they are all set to have $A_{t}=0$ (k=0,…,4) (“never treated”). In each dataset the $L_{k}$ (k=1,…,4) were then generated sequentially using the model for $L_{k}$ in Table 1, with $L_{k}$ being generated conditional on $L_{k-1}$, U and $A_{k}$. Event times were generated in each data set according to the conditional additive hazard model in (27). The true survival probabilities, and corresponding risk differences, under each treatment strategy (“always treated” and “never treated”) were then obtained using Kaplan‐Meier estimates.

Plots are used to present survival probability estimates and bias and efficiency results for the risk differences at time horizons τ in the range from 0 to 5. We also present results for the survival probabilities and risk differences at time horizons τ=1,2,3,4,5 in tables. Estimates are accompanied by Monte Carlo errors.

**FIGURE 6:**
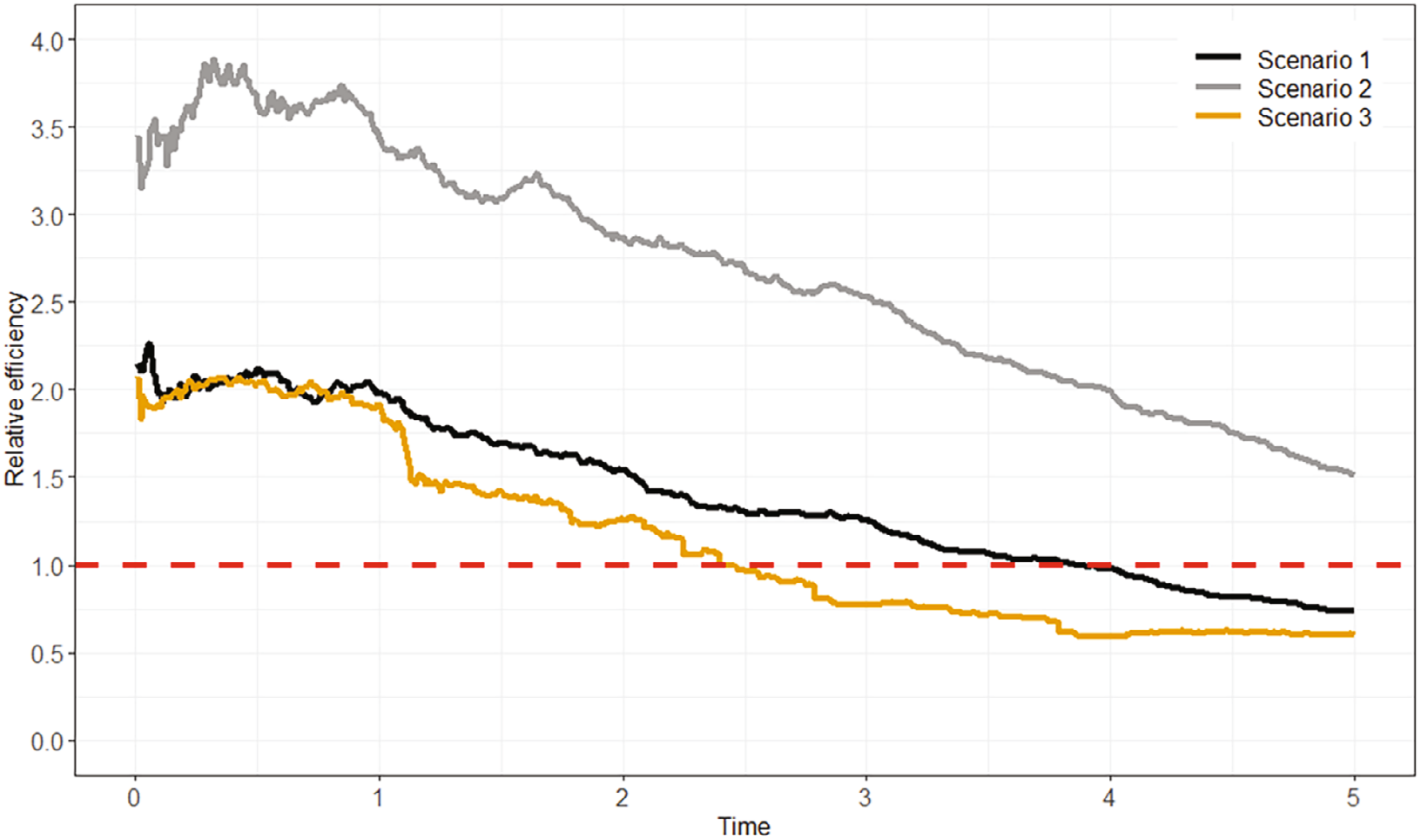
Simulation results: relative efficiency of the sequential trials analysis compared with the MSM‐IPTW analysis, defined as the inverse of the ratio of the empirical variances of the risk difference estimates at each time.

### 6.2 Results

**FIGURE 7:**
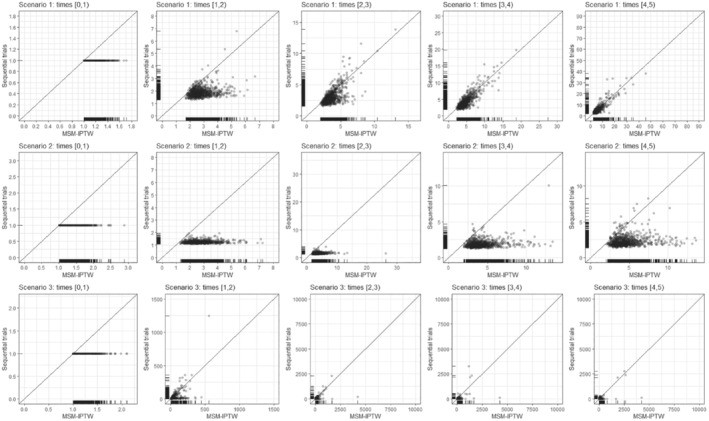
Simulation results: Plots of the largest weight (by time period) in each of the 1000 simulated data sets under the sequential trials analysis (IPACW) compared with the inverse probability weighted estimation of marginal structural model analysis. The weights are time‐dependent and change at event visit k=0,…,4. We obtained the largest weight in each time period, where in the sequential trials the time refers to time since the start of the trial. In the sequential trials analysis, the IPACW are equal to 1 up to time 1.

For the MSM‐IPTW approach we focus on the results obtained using the MSM conditional on $L_{0}$. Corresponding results for the MSM that is not conditional on $L_{0}$ are shown in the Supplementary Materials (Supplementary Figures 1 and 2). Figure 4 shows the estimated survivor curves under the “always treated” and “never treated” regimes under the three simulation scenarios, alongside the true curves. Figure 5 summarizes the corresponding results for bias in risk difference estimates. The results are summarised numerically at time points 1, 2, 3, 4, and 5 in Table 3. The relative efficiency of the sequential trials approach compared with the MSM‐IPTW approach is illustrated in Figure 6. Figure 7 shows plots of the largest weight used in the MSM‐IPTW and sequential trials analyses in each of the 1000 simulated datasets, by time‐period (because the weights are time‐dependent).

**TABLE 3:** Simulation results: Estimated survival probabilities (“Est”) under the “always treated” (S1) and “never treated” (S0) regimes and risk difference (RD) (mean over 1000 simulations) at time τ, with corresponding empirical SD (over 1000 simulations). Corresponding bias in the estimates ($\times 10^{4}$), accompanied by the MC error (×100).

		Time τ				
		1	2	3	4	5
MSM‐IPTW: scenario 1						
S1	Est (SD)	0.854 (0.021)	0.738 (0.027)	0.644 (0.030)	0.566 (0.031)	0.498 (0.031)
	Bias (MC error)	0.170 (0.066)	0.166 (0.084)	0.229 (0.094)	0.223 (0.097)	0.189 (0.099)
S0	Est (SD)	0.819 (0.015)	0.671 (0.019)	0.547 (0.024)	0.445 (0.029)	0.361 (0.033)
	Bias (MC error)	0.002 (0.047)	0.114 (0.061)	0.100 (0.076)	0.137 (0.091)	0.098 (0.105)
RD	Est (SD)	0.034 (0.026)	0.067 (0.033)	0.097 (0.040)	0.121 (0.044)	0.138 (0.047)
	Bias (MC error)	0.168 (0.082)	0.053 (0.106)	0.129 (0.126)	0.086 (0.139)	0.092 (0.148)
Sequential trials: scenario 1						
S1	Est (SD)	0.853 (0.015)	0.736 (0.019)	0.641 (0.022)	0.563 (0.024)	0.496 (0.027)
	Bias (MC error)	0.068 (0.047)	0.010 (0.059)	−0.068 (0.068)	−0.086 (0.076)	−0.037 (0.085)
S0	Est (SD)	0.819 (0.010)	0.669 (0.017)	0.544 (0.025)	0.442 (0.033)	0.359 (0.044)
	Bias (MC error)	−0.043 (0.031)	−0.085 (0.054)	−0.202 (0.079)	−0.191 (0.105)	−0.076 (0.140)
RD	Est (SD)	0.034 (0.018)	0.067 (0.027)	0.097 (0.036)	0.121 (0.044)	0.137 (0.055)
	Bias (MC error)	0.111 (0.058)	0.094 (0.085)	0.134 (0.112)	0.105 (0.140)	0.039 (0.173)
MSM‐IPTW: scenario 2						
S1	Est (SD)	0.855 (0.049)	0.739 (0.063)	0.648 (0.071)	0.573 (0.072)	0.506 (0.074)
	Bias (MC error)	0.333 (0.154)	0.343 (0.199)	0.569 (0.224)	0.934 (0.227)	0.933 (0.233)
S0	Est (SD)	0.819 (0.012)	0.671 (0.015)	0.547 (0.016)	0.445 (0.018)	0.361 (0.018)
	Bias (MC error)	0.018 (0.039)	0.123 (0.048)	0.133 (0.052)	0.112 (0.055)	0.077 (0.056)
RD	Est (SD)	0.036 (0.050)	0.069 (0.065)	0.100 (0.073)	0.128 (0.075)	0.145 (0.077)
	Bias (MC error)	0.315 (0.159)	0.219 (0.206)	0.436 (0.231)	0.823 (0.237)	0.856 (0.243)
Sequential trials: scenario 2						
S1	Est (SD)	0.852 (0.026)	0.735 (0.036)	0.640 (0.042)	0.564 (0.048)	0.498 (0.056)
	Bias (MC error)	−0.032 (0.083)	−0.125 (0.112)	−0.162 (0.132)	0.038 (0.150)	0.195 (0.178)
S0	Est (SD)	0.819 (0.007)	0.669 (0.012)	0.544 (0.017)	0.442 (0.020)	0.359 (0.024)
	Bias (MC error)	−0.030 (0.023)	−0.088 (0.039)	−0.184 (0.052)	−0.189 (0.063)	−0.128 (0.075)
RD	Est (SD)	0.033 (0.027)	0.066 (0.038)	0.096 (0.046)	0.122 (0.053)	0.140 (0.062)
	Bias (MC error)	−0.001 (0.086)	−0.036 (0.122)	0.022 (0.145)	0.227 (0.168)	0.324 (0.197)
MSM‐IPTW: scenario 3						
S1	Est (SD)	0.852 (0.023)	0.736 (0.034)	0.642 (0.042)	0.564 (0.046)	0.497 (0.049)
	Bias (MC error)	0.013 (0.074)	0.044 (0.108)	−0.007 (0.134)	0.005 (0.146)	0.001 (0.154)
S0	Est (SD)	0.820 (0.018)	0.674 (0.040)	0.558 (0.056)	0.460 (0.067)	0.382 (0.072)
	Bias (MC error)	0.100 (0.056)	0.478 (0.125)	1.165 (0.178)	1.631 (0.213)	2.181 (0.229)
RD	Est (SD)	0.032 (0.033)	0.062 (0.065)	0.084 (0.086)	0.104 (0.097)	0.115 (0.103)
	Bias (MC error)	−0.087 (0.106)	−0.434 (0.204)	−1.172 (0.271)	−1.626 (0.307)	−2.180 (0.327)
Sequential trials: scenario 3						
S1	Est (SD)	0.853 (0.017)	0.736 (0.027)	0.642 (0.044)	0.564 (0.060)	0.497 (0.060)
	Bias (MC error)	0.046 (0.053)	0.005 (0.087)	0.021 (0.138)	0.079 (0.189)	0.077 (0.191)
S0	Est (SD)	0.819 (0.013)	0.672 (0.036)	0.554 (0.063)	0.458 (0.080)	0.383 (0.091)
	Bias (MC error)	−0.005 (0.041)	0.215 (0.115)	0.775 (0.198)	1.427 (0.254)	2.344 (0.287)
RD	Est (SD)	0.033 (0.024)	0.064 (0.058)	0.088 (0.098)	0.106 (0.126)	0.114 (0.133)
	Bias (MC error)	0.051 (0.076)	−0.211 (0.182)	−0.754 (0.309)	−1.347 (0.399)	−2.267 (0.420)

In scenario 1 both methods give unbiased estimates of the risk difference at all time points. The sequential trials approach is more efficient up to time 4, after which the MSM‐IPTW approach is more efficient. The efficiency gain from the sequential trials approach is greatest at earlier time points and diminishes as time progresses. Figure 7 shows that the sequential trials approach tends to use less extreme weights (the IPACW) than the MSM‐IPTW approach at the earlier time points, but that by time 4 after potential treatment initiation the IPTW do not appear to be more extreme than than the IPACW. The relative efficiency results are a function of how the data are used in combination with modeling assumptions. Under the assumption that treatment effects on the hazard are the same across trials (which is true under our data generating mechanism), the sequential trials approach uses information about the treatment effect on the hazard in time period 0<t<1 from five trials, time period 1≤t<2 from four trials and so on. Only the first trial (starting at k=0) provides information about the time period 4≤t<5. The MSM‐IPTW analysis draws information on the impact of treatment in time period 4≤t<5 from individuals who initiated treatment at times 0,1,2,3,4, Table  1 in the Supplementary Materials shows the number of individuals observed at times t=0,1,2,3,4 under the two approaches. The number of individuals observed at time 4 is considerably greater under the MSM‐IPTW approach compared with the sequential trials approach.

In scenario 2, the sequential trials approach gives unbiased estimates of the risk difference at all time points. The MSM‐IPTW approach gives unbiased estimates up to around time 3, with the risk difference being slightly biased upwards after time 3. Figure 4 shows that the survivor curve in the “always treated” group is slightly biased upwards, which we also see in Table 3. The bias is small in magnitude but statistically significant based on 95% Monte Carlo confidence intervals. Figure 7 comparing the IPACW to the IPTW shows that the largest weights tend to be considerably higher in the MSM‐IPTW analysis compared with the sequential trials analysis. The difference between the IPACW and IPTW is much greater in scenario 2 compared with scenario 1, with the IPACW tending to be much less extreme than the IPTW at all time points. In scenario 2 the proportion of individuals initiating treatment at a given visit is low. Table S1 in the Supplementary Material shows that in scenario 2 the absolute numbers of individuals in the “always treated” group is low across all visits. The bias for MSM‐IPTW in scenario 2 is therefore attributed to the combination of large weights and finite sample bias due to small numbers of treated individuals. The relative efficiency plot in Figure 6 shows that the sequential trials analysis is more efficient than MSM‐IPTW at all time points. From Figure 4 and Table 3, we see that the variation in the estimates of the survival probabilities is much greater in MSM‐IPTW for the “always treated” regime. For the “never treated” regime the variation is smaller using the sequential trials approach up to between times 2 and 3, after which the variation in the MSM‐IPTW estimates is smaller.

In scenario 3, both methods give bias in the risk difference after time 1 (Figure 5). Figure 4shows that the survival probabilities for the “always treated” regime are estimated without bias, but the survival probabilities for the “never treated” regime have some upwards bias at later time points. The magnitude of the bias is small and similar under both methods. Figure 6 shows that the sequential trials analysis is more efficient up to around time 2.5, and the MSM‐IPTW analysis is more efficient thereafter. In this scenario the time‐varying covariate $L_{k}$ is strongly predictive of treatment $A_{k}$ (log odds ratio 3). Looking at the distribution of the largest weights in Figure 7 we see that after time 1 there are some very large weights under both methods. The largest weights tended to be higher in the MSM‐IPTW approach. The number of individuals observed at each time point is greater under scenario 3 than scenario 2. In this case therefore, the bias may be attributed to near violations of the positivity assumption.

Results are similar from the MSM‐IPTW analysis based on the MSM without conditioning on $L_{0}$ (Supplementary Material Figures 1 and 2). We also applied the analyses using truncated weights in which both IPTW and IPACW were truncated at the 95th percentile, which resulted in similar findings—see Supplementary Material Figures 3  and 4.

## 7 APPLICATION IN THE UK CF REGISTRY

### 7.1 Analysis

We apply the methods to the motivating application introduced in Section 2. Some initial data set‐up steps were taken as follows. Data are intended to be collected at annual visits. However, there are some visits considerably closer and wider apart. In order to structure the data into a form with regular visits for each individual, we omitted visits that were less than 6 months after the previous visit *and* where the previous and subsequent visit were less than 18 months apart. This follows what was done in previous work using the same data source.^54^ Where there was a period of more than 18 months after a given visit without the event or another visit being recorded, the individual was censored at the date of that visit plus 18 months, and any subsequent later visits excluded. There was a small amount of missing data in some of the time‐dependent covariates (∼10% missingness in covariates at the first visit). We used the last‐observation‐carried‐forward to address this. For the genotype variable “missing” was treated as a separate category.

The causal estimand was specified in Section 3.2, and is accompanied by a specification of the target trial and emulated trial in Table 1. The MSM‐IPTW analysis and the sequential trials analysis were both applied as outlined in Section 4 to estimate survival curves up to 11 years under the two treatment regimes of “always treated” or “never treated” with DNase, and corresponding risk differences. For the MSM‐IPTW analysis an individual's baseline visit (k=0) was defined as the first visit in the period 2008 to 2017 at which an individual meets the trial emulation eligibility criteria. For the sequential trials analysis individuals were assessed for eligibility at each visit from the baseline visit onwards, resulting in 10 possible trials.

For each estimation approach we obtained results using a Cox proportional hazards model and an Aalen additive hazards model (with time‐varying coefficients) for the MSMs. In the MSMs used for the MSM‐IPTW analysis the MSM included current treatment and treatment in the three prior years, and was conditional on the time‐fixed covariates and measures of the time‐varying covariates at the first visit, except visit year (see below). The MSM used in the sequential trials analysis included the same set of covariates as used in the MSM‐IPTW analysis, that is, the time‐fixed covariates and time‐varying covariates as measured at the visit at the start of each trial. The continuous covariates (age, FEV
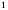
%, BMI *z*‐score) were modeled using restricted cubic splines (with three knots) in the MSMs. For the sequential trials analysis, we performed tests of whether the coefficients for treatment status differed by trial. There was no evidence of this and so we used a combined analysis across trials. In the analyses using the Cox proportional hazards model we assessed the proportional hazards assumption for the treatment variable(s). In the sequential trials analysis there was no evidence against the proportional hazards assumption for the treatment variable, whereas in the MSM‐IPTW analysis there was evidence against the proportional hazards assumption in a joint test for the four treatment variables in the model.

Logistic regression models were used to estimate stabilized weights for use in each analysis method. In the MSM‐IPTW approach the model used for the denominator of the weights included all time‐fixed covariates and the current values of the time‐dependent covariates. The model used for the numerator of the weights included the time‐fixed covariates and measures of the time‐varying covariates at the first visit (k=0). In these models, DNase status recorded at visit k is assumed to depend on FEV
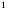
% and BMI z‐score recorded at visit k−1, and on other time‐dependent variables as recorded at visit k. In the sequential trials analysis we estimated separate IPACW for artificial censoring due to switching from $A_{k}=0$ to $A_{k+1}=1$ and for switching from $A_{k}=1$ to $A_{k+1}=0$. In the simulation we assumed that once an individual initiates treatment they always continue treatment. However, in this application some individuals also stop treatment. The models for the weights included the same variables as included in the weights for the MSM‐IPTW analysis. The continuous variables (Age, FEV
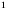
%, BMI *z*‐score, visit year) were modeled using restricted cubic splines (with three knots) in the models for the weights.

For both approaches we also estimated stabilized weights for censoring due to loss‐to‐follow‐up. These included the same variables as used in the treatment‐related weights, with the difference that the censoring weights models for the probability of being censored between visits k and k+1 included measures of FEV
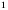
% and BMI *z*‐score made at visit k, rather than the measures from the previous visit. This is because the censoring indicator at visit k refers to censoring before visit k+1, whereas the treatment indicator at visit k refers to treatment status since the previous visit.

We wished to estimate the same marginal survival probabilities, and hence risk differences, using each analysis method. Both methods include conditioning on covariates measured at the first visit at the start of each trial. We are therefore able to standardize to any appropriate population. As discussed above and in Section 3.2, for this analysis we standardize to the population of individuals meeting the trial emulation eligibility criteria in 2018. Visit year was included in the weights models but not in the set of variables included in the MSMs. This is because there can only be little information about long term follow‐up for individuals in the later years, which results in poor convergence in some models if visit year is included in the MSMs. There are therefore slight differences between the population to which the MSM‐IPTW analyses refer and that to which the sequential trials analyses refer, in terms of the marginal distribution of years at t=0 from 2008 to 2017. We consider this to be a minor difference that is unlikely to result in clinically important differences between the results from the two approaches.

Bootstrapping was used to obtain 95% confidence intervals for the estimated survival curves under the two treatment regimes of “always treated” or “never treated” with DNase and the corresponding risk differences, using 1000 bootstrap samples and the percentile method.

### 7.2 Results

The trial emulation eligibility criteria were met by 3855 individuals at at least one visit during 2008 to 2017. Among these individuals there were 338 events (266 deaths and 72 transplants). Of the 3517 individuals who did not have the event, 1780 (51%) were administratively censored (defined as having the expected number of visits given their first year of meeting the trial emulation eligibility criteria, assuming one visit each year) and the remainder were censored due to loss‐to‐follow‐up (which included individuals censored after a gap of more than 18 months without a visit). The characteristics of the individuals at the first visit at which the trial emulation eligibility criteria were met (time k=0) are summarised in Table 4.

**TABLE 4:** Application in the UK CF Registry data: Summary of individual characteristics at the first visit at which trial emulation eligibility criteria were met.

Variable	Mean (SD)	Median (IQR)
Age (years)	24.32 (11.56)	21.6 (14.39,30.54)
FEV 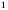 %	76.12 (21.93)	78.8 (62.2,92.38)
BMI *z*‐score	0.15 (1.22)	0.14 (−0.6,0.96)
	N	%
**Sex**		
Male	2120	(55%)
Female	1735	(45%)
**Genotype class**		
High	2586	(67%)
Low	596	(15%)
Not assigned	594	(15%)
Missing	79	(2%)
**Presence of airway infections in the past 12 months**		
*Staphylococcus aureus* or Methicillin‐resistant *Staphylococcus aureus* (MRSA)	1676	(43%)
*Pseudomonas aeruginosa* (or use of nebulized antibiotics)	2453	(64%)
*Burkholderia cepacia*	113	(3%)
*Non‐tuberculous mycobacteria* (NTM)	162	(4%)
**Presence of complications**		
CF‐related diabetes	707	(18%)
Pancreatic insufficiency	3028	(79%)
**Use of other treatments in the past 12 months**		
Other mucoactive treatments (hypertonic saline, mannitol or acetylcysteine)	554	(14%)
CFTR modulators (ivacaftor, lumacaftor/ivacaftor, or tezacaftor/ivacaftor)	54	(1%)
Oxygen therapy	153	(4%)
Hospitalization (not for IV antibiotics)	145	(4%)
Days receiving IV antibiotics		
0	2315	(60%)
1‐14	640	(17%)
15‐28	329	(9%)
29‐42	227	(6%)
>42	344	(9%)

Table 5 summarizes the number of individuals contributing to the MSM‐IPTW and sequential trials analyses and their treatment status. The trial emulation eligibility criteria were first met in 2008 (the first year we considered) by 1729 individuals (45%). In the sequential trials approach many individuals contribute to trials starting at multiple time points such that 18 439 rows contribute information at the start of trials (Table 5B). At visit k=10 (ie, 10 years after first meeting the trial emulation eligibility criteria), there remain 643 individuals in the MSM‐IPTW analysis but only 241 rows in the sequential trials analysis, due to the artificial censoring in the sequential trials approach. At the first visit at which trial emulation eligibility criteria were met, 612 initiated DNase treatment (16%). Of those who initiated DNase at this first eligible visit, 69% used DNase at all visits, and of those who did not initiate DNase at the first eligible visit 53% were non‐users at all visits. In the MSM‐IPTW analysis the proportion of treated individuals is similar across visits, ranging from 15% to 17%. In the sequential trials analysis the proportion of treated individuals at a given visit is equivalent to the proportion “always treated” up to that visit (due to the artificial censoring), and this percentage increases over time from 15% at visit k=0 to 22% at visit k=10.

**TABLE 5:** Summary of number of individuals contributing to the MSM‐IPTW and sequential trials analyses and numbers untreated and treated: (A) by year, (B) by visit number.

(A) By year							
	MSM‐IPTW			Sequential trials			
Year	Number observed	Untreated	Treated	Number observed	Untreated	Treated	
2008	1729	1452 (84%)	277 (16%)	1729	1452 (84%)	277 (16%)
2009	537	440 (82%)	97 (18%)	1908	1599 (84%)	309 (16%)
2010	352	285 (81%)	67 (19%)	2004	1637 (82%)	367 (18%)
2011	359	310 (86%)	49 (14%)	2039	1731 (85%)	308 (15%)
2012	264	234 (89%)	30 (11%)	2040	1728 (85%)	312 (15%)
2013	179	156 (87%)	23 (13%)	1916	1652 (86%)	264 (14%)
2014	152	127 (84%)	25 (16%)	1866	1601 (86%)	265 (14%)
2015	123	100 (81%)	23 (19%)	1779	1519 (85%)	260 (15%)
2016	90	79 (88%)	11 (12%)	1629	1426 (88%)	203 (12%)
2017	70	60 (86%)	10 (14%)	1529	1325 (87%)	204 (13%)

(B) By visit number k						
	MSM‐IPTW				Sequential trials		
Visit	Number observed	Untreated	Treated		Number observed	Untreated	Treated
0	3855	3243 (84%)	612 (16%)		18439	15670 (85%)	2769 (15%)
1	3466	2901 (84%)	565 (16%)		16889	14321 (85%)	2568 (15%)
2	3121	2609 (84%)	512 (16%)		12302	10347 (84%)	1955 (16%)
3	2821	2350 (83%)	471 (17%)		8958	7408 (83%)	1550 (17%)
4	2500	2101 (84%)	399 (16%)		6308	5196 (82%)	1112 (18%)
5	2205	1851 (84%)	354 (16%)		4380	3564 (81%)	816 (19%)
6	1870	1565 (84%)	305 (16%)		2929	2335 (80%)	594 (20%)
7	1570	1308 (83%)	262 (17%)		1899	1490 (78%)	409 (22%)
8	1246	1036 (83%)	210 (17%)		1154	882 (76%)	272 (24%)
9	972	824 (85%)	148 (15%)		609	472 (78%)	137 (22%)
10	643	548 (85%)	95 (15%)		241	187 (78%)	54 (22%)

**FIGURE 8:**
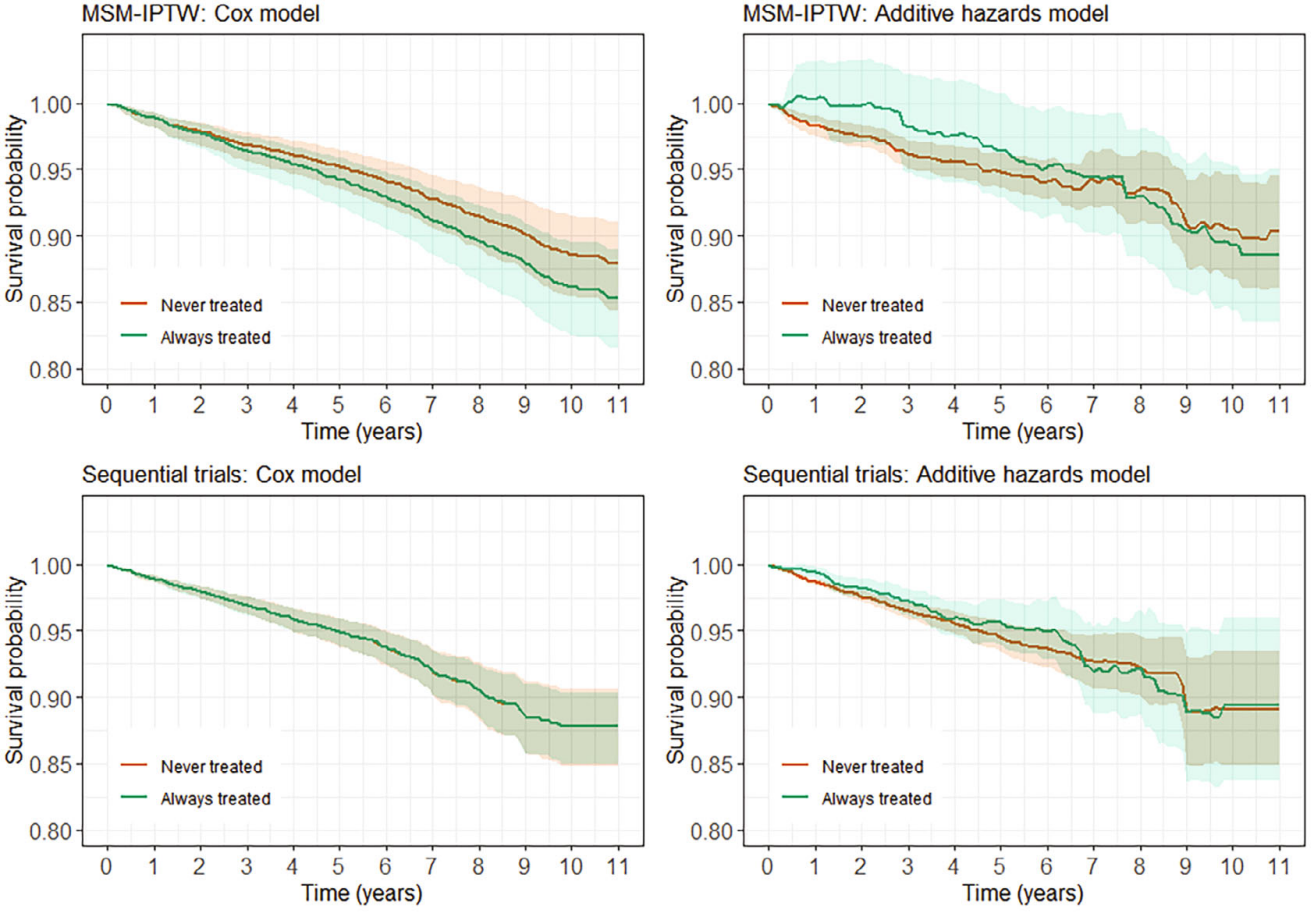
Estimated survival curves under the “always treated” and “never treated” with DNase strategies from the MSM‐IPTW and sequential trials analyses. The shaded areas show the 95% confidence intervals.

**FIGURE 9:**
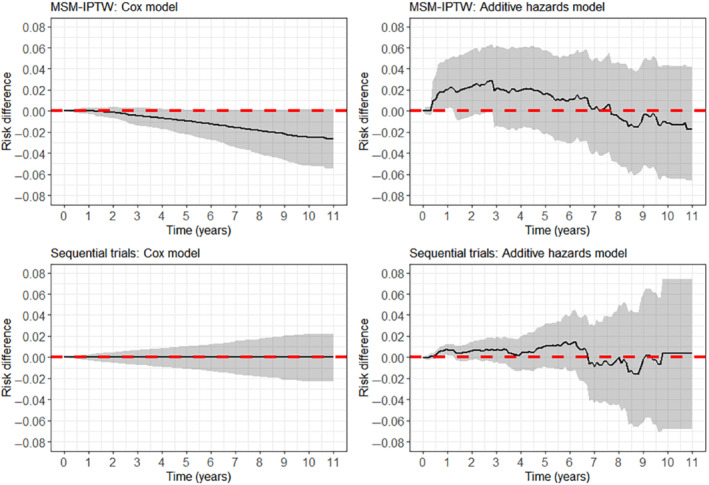
Estimated risk difference (“always treated” vs “never treated” with DNase) from the MSM‐IPTW and sequential trials analyses. The shaded area shows the 95% confidence intervals.

Figure 8 shows the estimated survivor curves from the MSM‐IPTW and sequential trials analyses, using Cox proportional hazards models and additive hazards models for the MSMs. The corresponding estimated risk differences are shown in Figure 9, where a risk difference greater than 1 indicates better survival in the ‘always treated’.

None of the results provide evidence that DNase use affects the outcome, either in the short or long term. In the MSM‐IPTW analysis using a Cox proportional hazards model, the survival curve for the “always treated” regime is consistently below that for the “never treated” regime, with the differences increasing over time. In the sequential trials analysis using the Cox model the survival curves in the two treatment groups are very similar over time. Using the additive hazards model the survival curve for the “always treated” regime is above that for the “never treated” regime for the first few years, more so in the MSM‐IPTW results, before the curves cross. As we would expect, the 95% CIs from using the Cox model are narrower than those from the additive hazards model. The 95% CIs are generally narrower under the sequential trials approach, except when using the additive hazards models in the later part of follow‐up. Figure S5 shows a plot of the distribution of the weights used under the two analysis approaches. This shows that a large number of rows are assigned a weight of 1 in the sequential trials analysis. This is because in the sequential trials data the first row corresponding to each trial has a weight of 1. There are no extreme weights in the MSM‐IPTW analysis, but the distribution is less highly concentrated around 1.

## 8 DISCUSSION

In this paper we have focused on estimating the effects of longitudinal treatment regimes on survival, using observational data subject to time‐varying confounding of the treatment‐outcome association. We have compared the MSM‐IPTW approach with the sequential trials approach. A key contribution has been to specify causal estimands that can be identified using the sequential trials approach, and we showed how the previously described implementations can be shown to estimate underlying MSMs for counterfactual outcomes under certain assumptions. We compared the MSM‐IPTW and sequential trials approaches in terms of how the data are used, the form of the MSMs, how time‐dependent confounding is addressed through the analysis, and when they identify the same parameters.

For time‐to‐event outcomes, estimands based on contrasts between risks rather than contrasts between hazards have been shown to have better causal interpretations. We outlined how the outputs from the sequential trials analysis and the MSM‐IPTW analysis can be used to obtain marginal absolute risk estimates using empirical standardization, and hence to obtain marginal risk differences or ratios. Care should be taken over defining the population to which marginal risk estimates refer. Applications of the sequential trials approach in the literature have tended to focus on presentation of hazard ratio estimates. As well as giving hazard ratio estimates, Hernan et al^4^ presented estimated survivor curves under the treated and untreated regimes. However, it was not clear how the survival curves were obtained or what population they referred to. Cole et al,^26^ Garcia‐Albinez et al,^18^ and Caniglia et al^13^ also obtained survival curves using the sequential trials approach but similarly the methods were not described in detail. In this paper, we have provided clear guidance on how to obtain estimated survival curves from the MSM‐IPTW and sequential trials approaches.

A key difference between the MSM‐IPTW approach and the sequential trials approach is that the MSM‐IPTW approach enables estimation of risks under any longitudinal treatment regime, whereas the sequential trials approach focuses only on “always treated” and “never treated” regimes. However, the sequential trials approach could be extended to consider different longitudinal treatment regimes, with individuals being censored when they deviate from a specified regime. Conversely, a modified version of the MSM‐IPTW approach that focuses only on “always treated” and “never treated” regimes could also be used. This would involve censoring individuals when they deviate from the “always treated” or “never treated” strategies, and applying weights as in the sequential trials approach. Such an approach has been referred to as a “clone‐censor‐weight” approach, and has more commonly been used in settings in which one of the treatment strategies is implemented within a grace period,^55^ but has not to our knowledge been explicitly linked to use of MSMs. If this approach used baseline covariate adjustment instead of weighting, then the difference between this and the sequential trials approach would only be a matter of the use of several trials from new time origins in the sequential trials approach, which would typically be accompanied by assumptions about certain parameters being common across trials.

van der Laan et al^56^ and Petersen and van der Laan^57^ introduced history‐adjusted MSMs (HA‐MSM), which are an extension of MSMs and connected to both approaches considered in this paper. In a HA‐MSM, an MSM is used from a series of new time points (visits) in a study, with the MSM being conditional on covariates and treatment history up to that time, and assuming a common MSM across time points. Estimation of the MSMs is using IPTW. As well as being an extension of the MSM‐IPTW approach, there is a clear connection between HA‐MSMs and the sequential trials approach. Hernan and Robins^15^ referenced HA‐MSMs in their paper describing the target trial framework when noting that eligibility criteria could be applied at multiple time points. A distinction between HA‐MSMs and the sequential trials approach is that the sequential trials approach restricts to individuals who have not yet started the treatment at each new time origin, whereas the HA‐MSM approach conditions on past treatment in the MSM. Like in MSMs, the HA‐MSM approach considers all longitudinal treatment regimes, in contrast to the sequential trials approach, which focuses only on two treatment regimes. An example of the use of HA‐MSMs in the context of a survival outcome was given by Bembom et al.^58^ However, the HA‐MSM approach does not yet appear to have been much used in practice, perhaps due to initial criticisms of the method.^59^, ^60^ The HA‐MSM approach requires assumptions about both how the history of treatment affects the hazard and how models can be combined across time origins, and therefore stronger assumptions overall than are required by either the MSM‐IPTW approach or the sequential trials approach. As noted in Section 1, the sequential trials approach is also closely related to the sequential stratification method described by Schaubel et al,^7^, ^8^ in which individuals initiating treatment at a given time point are matched to untreated individuals in a sequential manner over time. The analysis results in an estimate of the treatment effect on the treated, that is, it targets a different causal estimand than that which we focus on in this paper. An area for future work is to further discuss the different sequential methods for causal inference for survival outcomes, including those summarized in the review by Thomas et al.,^6^ but also extending to HA‐MSMs, in particular to compare the estimands that they focus on and their assumptions.

We focused in this paper on a setting in which the trials used in the sequential trials approach have different lengths of follow‐up, with trials starting at later times having shorter follow‐up. It would also be possible to focus only on trials with the same fixed length of follow‐up. This depends on the total length of follow‐up available in the data, and on the time horizons of interest for the risk differences being estimated. For example, in the simulation we consider a situation with 5 visit times. Had we been interested in a risk difference for a time horizon of τ=2, we could have used sequential trials of equal length starting at times 0, 1, 2, and 3. For the MSM‐IPTW analysis, the question would arise as to whether to focus on an analysis starting from time 0, or starting from time 3, say. Using sequential trials of equal length of follow‐up would make it possible to allow a separate baseline hazard for each trial covering the full length of follow‐up needed for the risks of interest, which would allow a choice as to which baseline hazard could be used to obtain risk estimates.

There are important considerations of timescale in the methods described in this paper. In our motivating example, the first visit (k=0) was defined as occurring at the first time during the observation period at which an individual met the trial emulation eligibility criteria. We included both age and calendar year in the set of covariates controlled for in the analysis. An alternative in this example would have been to define k=0 as a particular calendar year (e.g. 2008), with people then entering the cohort in later years at which they first meet the trial emulation eligibility criteria. In this example we believe the choice between these two options will not have any important consequences for the findings as we control for calendar year in the analysis. In the MSM‐IPTW approach our definition of k=0 enables more people to be included in the analysis than would have been included if we defined k=0 as 2008. In some other clinical contexts a person's time since diagnosis will be another important aspect to consider. In our example a person's time since diagnosis was not a major consideration as the majority of people with CF are diagnosed soon after birth. When using longitudinal data and methods such as those focused on in this paper, different aspects of time should be carefully considered, and the choice of time scale for the analysis clearly defined.

We compared the MSM‐IPTW and sequential trials approaches using a simulation study, focusing on bias and efficiency. We found that the sequential trials approach can be more efficient than the MSM‐IPTW approach for estimating risk differences, but that this is not true at all time points: for risks at time points corresponding to longer follow‐up the MSM‐IPTW approach can be more efficient. We linked the efficiency gains from the sequential trials approach to the use of less extreme weights (IPACW) compared with the IPTW. The efficiency gains from the sequential trials approach at some time points arise due to assumptions about equality of the treatment effect across different trials and assumptions about how much covariate history at the start of each trial needs to be conditioned on in the MSM for the hazard in order to control for confounding. Throughout the paper we discussed the methods in terms of Cox proportional hazard models or Aalen additive hazard models for the MSM. Previous work in this context has focused on Cox models for the MSM. We have shown that the additive hazard model can have some advantages in terms of relaxing assumptions—for example, it naturally allows the hazard to depend on treatment duration in the sequential trials approach. Use of the additive hazard model was also advantageous in the simulation study as it ensured that the analysis models used in both the MSM‐IPTW approach and in the sequential trials approach could both be correctly specified, enabling a fair comparison of the methods. There have been few simulation comparisons of causal methods for time‐to‐event outcomes due to the challenges of making fair comparisons. As noted earlier, Karim et al^21^ compared the MSM‐IPTW and sequential trials approaches but focused on estimation of hazard ratios and compared efficiency of marginal and conditional hazard ratios, which are not the same quantity. In further work it would be of interest to use a similar simulation approach to compare other methods, including the other sequential methods noted above. It would also be of interest to compare the two approaches when the MSMs used are mis‐specified, and to investigate the impacts of model mis‐specification.

The methods were applied to investigate the effect of the treatment DNase for people with CF on the composite outcome of death or transplant using longitudinal observational data from the UK CF Registry. We did not find any evidence of a causal treatment effect using either the MSM‐IPTW approach or the sequential trials approach. In recent work, longitudinal data from the UK CF Registry was used to estimate the longer term causal effect of DNase on the non‐time‐to‐event outcomes of lung function and number of days of intravenous antibiotic medication using MSM‐IPTW, g‐computation and g‐estimation.^38^, ^39^ This work found evidence of a benefit of DNase for people with low lung function, but not for those whose lung function was higher. Seaman et al^54^ illustrated the structural nested cumulative survival time approach with an investigation of the effect of DNase use on survival using UK CF Registry data, and also did not find evidence of a treatment effect. Sawicki et al^61^ used longitudinal data from the US Cystic Fibrosis Foundation patient Registry to investigate the effect of DNase treatment on the risk of death in the next year using pooled logistic regression analyses, finding evidence of a reduced risk of death with DNase use. Limitations of our analysis include that DNase is used by a large proportion of the population from a young age, and we focused on individuals initiating DNase aged 12 and older, as there are few deaths in younger individuals. It is possible that patients initiating DNase at older ages have particular characteristics that are also associated with survival, and which we did not fully capture in the set of adjustment variables used in our analysis. The number of events in our data was relatively low, which at least in part explains the wide uncertainty on our estimates. Our analysis included a relatively large number of adjustment variables compared to the number of events, which included several continuous variables, and we did not include any interaction terms in the weights models or the MSMs. In further work it would be of interest to investigate data‐adaptive methods for flexible adjustment for the time‐dependent confounders. Another important consideration in interpreting our results is that the UK CF Registry records DNase prescription, but adherence to the treatment is unknown and may be low and dependent on individual characteristics. The target trial we considered was pragmatic and did not specify the treatment dose or regimen, as reliable information on this is not available in the observational data used to emulate the trial. Under both treatment strategies use of other treatments was not specified, and people with CF typically use a number of treatments. Therefore the total effect of DNase may be partly through its effect on use of other treatments. In further work it would be of interest to use Registry data to investigate the impact of DNase in combination with other commonly used treatments and also the impact removing DNase treatment, particularly in light of new disease‐modifying treatments that have been introduced for CF in recent years. The MSM‐IPTW and sequential trials methods as described in this paper assume that information on treatment use and time‐dependent confounders is observed at regular visit times. This was the foundation of the data structure in the motivating example, which is based on annual review clinic visits conducted approximately 12 months apart. However in many settings, for example, in routinely collected data, data are not collected at a regular frequency, and dates of exact treatment initiation (and cessation) may be recorded. Such treatment data are now available for the UK CF Registry. An important area for further research is to consider the extension of the methods discussed in this paper to accommodate data in which information on treatment and/or confounders are not recorded at regular visit times.

In Section 3 we linked the causal estimand, a marginal risk difference, to a target trial and we outlined a protocol for a target trial for our motivating example. It could be said that using the target trial framework is not needed if the causal estimand is precisely defined. However, we found that specifying a target trial alongside the causal estimand helps to clarify its interpretation, particularly what the treatment strategies are and which population the estimand refers to. Use of the target trial framework also makes the aims and methods accessible to audiences unfamiliar with the counterfactual framework. The concept of linking causal inference investigations using observational data to a hypothetical trial has a long history, for example summarised by Hernan and Robins (section 3.6),^52^ and includes Robins'^62^ extension of this concept to longitudinal treatment strategies. There is strong link between the target trial framework of Hernan and Robins (2016)^15^ and the general roadmap for causal inference proposed by Petersen and van der Laan,^63^ though the latter places an emphasis on formal specification of the causal estimand using counterfactual notation.

In summary, the MSM‐IPTW and sequential trials approaches can both be used to estimate causal effects of time‐varying treatments using observational data, controlling for time‐dependent confounding. The sequential trials approach is appealing in that it enables partial regression adjustment for time‐varying confounders, thereby lessening reliance on possibly highly variable inverse probability of treatment weights. It is important to recognise that this comes at the price of needing to make certain modelling assumptions that are not required when using MSM‐IPTW. Both approaches rely on modeling assumptions. The MSM that is used in the MSM‐IPTW approach considers all treatment patterns, which makes it prone to mis‐specification. The sequential trials approach focuses only on two regimes, meaning that fewer assumptions need to be made about how the hazard depends on the history of treatment. However, the sequential trials approach is prone to model misspecification due to the use of a combined analysis across trials and the need to model treatment effects conditional on the covariate history, though the latter is also required in MSM‐IPTW using stabilized weights that conditional on baseline covariates. The assumptions made in a sequential trials analysis combined across trials are arguably stronger than those required in the MSM‐IPTW approach and the consequences of model mis‐specification could be more severe. The difference between the two approaches could be considered in terms of the sequential trials approach borrowing information or smoothing across populations, whereas the MSM‐IPTW approach borrows information across treatment regimes. Even if the MSM used in the MSM‐IPTW approach is mis‐specified, it is unlikely to induce an impression of treatment effect when there is none, and the results may be interpreted as an average effect over multiple regimes. The assumptions made in the sequential trials approach can be assessed by inclusion of interaction terms between trial and treatment status. A number of variations on, or extensions to, the two approaches were noted above, including of the sequential trials approach to consider more than two treatment regimes, and of the MSM‐IPTW approach to restrict focus to just the “always treated” and “never treated” regimes. The potential to use the MSM‐IPTW and sequential trials approaches in different ways presents areas for future investigation, including comparisons of their assumptions and performance.

## FUNDING INFORMATION

Ruth H. Keogh is funded by a UK Research & Innovation Future Leaders Fellowship (MR/S017968/1), Jon Michael Gran by the Research Council of Norway (Grant No. 273674), Shaun R. Seaman by MRC programme grant MC_UU_00002/10, and Gwyneth Davies by a UK Research & Innovation Future Leaders Fellowship (MR/T041285/1).

## Supporting information



Supplementary Material

## Data Availability

This work used anonymized data from the UK CF Registry, which has Research Ethics Approval (REC ref: 07/Q0104/2). The use of the data was approved by the Registry Research Committee. Data are available following application to the Registry Research Committee. https://www.cysticfibrosis.org.uk/the‐work‐we‐do/uk‐cf‐registry/apply‐for‐data‐from‐the‐uk‐cf‐registry. The authors thank people with CF and their families for consenting to their data being held in the UK CF Registry, and NHS teams in CF centers and clinics for the input of data into the Registry.
