# Supplementary material for: Causal inference in survival analysis using longitudinal observational data: Sequential trials and marginal structural models
Source: Stat Med. 2023 Apr 22;42(13):2191–225. doi: 10.1002/sim.9718 (PMC7614580; doi:10.1002/sim.9718)
Supplement: Supplementary file 1 — Supplementary Material [file SIM-42-2191-s001.pdf]

Causal inference in survival analysis using longitudinal observational data: Sequential trials and marginal structural models

Supplementary Material

1 Assumptions

The no interference assumption is that the counterfactual event time for a given individual, $T^{\underline{a}_0}$, does not depend on the treatment received by any other individuals. The positivity assumption is that each individual has a strictly non-zero probability of receiving each given pattern of treatments over time. Consistency means that an individual's observed outcome is equal to the counterfactual outcome when the assigned treatment pattern is set to that which was actually received, $T_i = T_i^{\underline{A}_{0,i}}$. The conditional exchangeability assumption can be expressed formally as $T^{\bar{A}_{k-1},\underline{a}_k} \perp\!\!\!\perp A_k|\bar{A}_{k-1}, \bar{L}_k, T \geq k$ for all feasible $\underline{a}_k$, where $T^{\bar{A}_{k-1},\underline{a}_k}$ denotes the counterfactual event time had an individual followed their observed treatment pattern up to time $k-1$, $\bar{A}_{k-1}$, and had their treatments been set to $\underline{a}_k$ from time k onwards, given survival to time k. The conditional exchangeability assumption means that among individuals who remain at risk of the event at time k, the treatment A_k received at time k may depend on past treatment and covariates $\bar{A}_{k-1}$ and $\bar{L}_k$, but that, conditional on these, it does not depend on the remaining lifetime that would apply if all future treatments were set to any particular values $\underline{a}_k$.

2 Inverse probability weights

2.1 MSM-IPTW

To estimate MSMs using IPTW, the weight at time t for individual i is the inverse of their probability of their observed treatment pattern up time time t given their time-dependent covariate history (Cole and Hernán, 2008, Daniel et al., 2013):

$$W_i(t) = \prod_{k=0}^{\lfloor t \rfloor} \frac{1}{\Pr(A_k = A_{k,i}|\bar{L}_{k,i}, \bar{A}_{k-1,i}, T \geq k)} \tag{1}$$

Some individuals can have very large weights, which can results in the parameters of the MSM being estimated very imprecisely, and therefore stabilized weights are typically used. The stabilized weight for individual i is:

$$SW_i(t) = \prod_{k=0}^{\lfloor t \rfloor} \frac{\Pr(A_k = A_{k,i}|\bar{A}_{k-1,i}, T \geq k)}{\Pr(A_k = A_{k,i}|\bar{L}_{k,i}, \bar{A}_{k-1,i}, T \geq k)} \tag{2}$$

The model in the numerator of the stabilized weights can also include covariates measured at time 0, $\bar{L}_0$:

$$SW_i(t) = \prod_{k=0}^{\lfloor t \rfloor} \frac{\Pr(A_k = A_{k,i}|\bar{A}_{k-1,i}, \bar{L}_{0,i}, T \geq k)}{\Pr(A_k = A_{k,i}|\bar{L}_{k,i}, \bar{A}_{k-1,i}, T \geq k)}, \tag{3}$$

in which case the MSM must also condition on these variables, as noted in the main text.

The models used in the weights can be estimated using logistic regression, and they may be fitted using all visits combined or separately by visit.

When the MSM for the hazard is a Cox model, the integrated (cumulative) baseline hazards required to obtain estimates of risk can be estimated using an IPTW Breslow's estimator (Main text Section 4.2). The estimate of the cumulative baseline hazard at time t is therefore given by

$$\widehat{H}_0(t) = \sum_{t_j \leq t} \frac{d_j}{\sum_{l \in R_j} w_l(t_j) e^{\hat{\beta}_A^\top \bar{a}_{\lfloor t_j \rfloor}}} \tag{4}$$

where the sum is over event times $t_j \leq t$, R_j denotes the risk set at time t_j, d_j denotes the number of events at time t_j (with $d_j = 1$ if there are no tied event times), and $w_l(t_j)$ denotes the IPTW for individual $l \in R_j$ at time t_j.

2.2 Sequential trials

For individuals in the trial starting at visit k but who do not initiate treatment at that time ($\bar{A}_k = 0$) the weight at time $t - k$ from the start of the trial is

$$W_{i,k}^0(t-k) = \prod_{j=k+1}^{k+\lfloor t \rfloor} \frac{1}{\Pr(A_j = 0 | \bar{L}_{j,i}, \bar{A}_{(k,j-1),i} = 0, T \geq k)} \tag{5}$$

where $\bar{A}_{(k,j-1)} = \{A_k, A_{k+1}, \ldots, A_{j-1}\}$ denotes the treatment status from visit k to visit $j-1$. For those who initiate treatment at the start of trial k ($\bar{A}_{k-1} = 0, A_k = 1$) the weight is

$$W_{i,k}^1(t-k) = \prod_{j=k+1}^{k+\lfloor t \rfloor} \frac{1}{\Pr(A_j = 1 | \bar{L}_{j,i}, \bar{A}_{k-1,i} = 0, \bar{A}_{(k,j-1),i} = 1, T \geq k)} \tag{6}$$

In the trial starting at visit k, the weights are equal to 1 for all individuals between time k and $k+1$, i.e. up to follow-up time 1 in trial k.

The corresponding stabilized weights are

$$SW_{i,k}^0(t-k) = \prod_{j=k+1}^{k+\lfloor t \rfloor} \frac{\Pr(A_j = 0 | \bar{L}_{k,i}, \bar{A}_{(k,j-1),i} = 0, T \geq k)}{\Pr(A_j = 0 | \bar{L}_{j,i}, \bar{A}_{(k,j-1),i} = 0, T \geq k)} \tag{7}$$

and

$$SW_{i,k}^1(t-k) = \prod_{j=k+1}^{k+\lfloor t \rfloor} \frac{\Pr(A_j = 1 | \bar{L}_{k,i}, \bar{A}_{k-1,i} = 0, \bar{A}_{(k,j-1),i} = 1, T \geq k)}{\Pr(A_j = 1 | \bar{L}_{j,i}, \bar{A}_{k-1,i} = 0, \bar{A}_{(k,j-1),i} = 1, T \geq k)} \tag{8}$$

where the probability in the numerator is conditional on the covariates $\bar{L}_k$ observed at the start of trial k.

The models used in the weights can be estimated using logistic regression, and they may be fitted using all visits combined or separately by visit, and across all trials combined or separately by trial.

3 Compatibility of differently specified MSMs

In Section 4 in the main text we considered MSMs based on Cox models or on additive hazard models. In this section we show that one can use an additive hazard model for the MSM for

the sequential trials analysis (equation (14)) (with common parameters assumed across trials) and an additive hazard model for the MSM used in the MSM-IPTW approach (equation (5)), and that both MSMs can be correctly specified. However, if we use a Cox model for the MSM for the sequential trials analysis (equation (13)) and a Cox model for the MSM used in the MSM-IPTW approach (equation (5)), then both MSMs cannot simultaneously be correctly specified in general. This is because the parameters of additive hazard models are collapsible, whereas hazard ratios in the Cox model are non-collapsible. Martinussen and Vansteelandt (2013)Martinussen and Vansteelandt (2013) explained the implications of collapsibility for the use of Aalen additive hazards models and Cox models in the context of estimating the causal effect of a point treatment on survival. Keogh et al. (2021)Keogh et al. (2021) extended to a longitudinal setting similar to that considered in this paper. Here we outline some key points in the context of the simple setting depicted in Figures 2 and 3 in the main text.

For the MSM-IPTW analysis in this section we focus on an MSM that is not conditional on L_0, as in equations (4), (5). The MSMs used in a MSM-IPTW analysis and in the sequential trials analysis may be considered to be consistent with each other if there exists an underlying fully conditional hazard model that implies both the MSM used in MSM-IPTW and the MSM used in the sequential trials approach, which is conditional on covariates measured at the start of each trial. As in Section 5.2 in the main text, for illustration we consider estimating $\Pr(Y_1^{a_0=a}=0)$ (or $\Pr(T^{\underline{a}_0=a}>1)$) and $\Pr(Y_2^{\bar{a}_1=a}=0)$ (or $\Pr(T^{\underline{a}_1=a}>2)$). First consider a fully conditional additive hazard model of the form

$$h(t|\bar{A}_{\lfloor t\rfloor},\bar{L}_{\lfloor t\rfloor})=\alpha_0(t)+\alpha_A(t)A_{\lfloor t\rfloor}+\alpha_L(t)L_{\lfloor t\rfloor}. \tag{9}$$

Under this model the conditional survival probability at time 1 is

$$\Pr(Y_1=0|A_0,L_0)=\exp\left(-\int_0^1(\alpha_0(u)+\alpha_A(u)A_0+\alpha_L(u)L_0)du\right) \tag{10}$$

and the marginal probability of survival to time 1 is

$$\begin{aligned}\Pr(Y_1^{A_0=a}=0)&=e^{-\int_0^1(\alpha_0(u)+\alpha_A(u)A_0)du}\Pr(L_0=0)+e^{-\int_0^1(\alpha_0(u)+\alpha_A(u)A_0+\alpha_L(u))du}\Pr(L_0=1)\\&=e^{-\int_0^1(\alpha_0^*(u)+\alpha_A(u)A_0)du},\end{aligned} \tag{11}$$

where $\int_0^1\alpha_0^*(u)du=\int_0^1\alpha_0(u)du+\log\{\Pr(L_0=0)+e^{-\int_0^1\alpha_L(u)du}\Pr(L_0=1)\}$. This expression is in the form of the survival probability from a marginal additive hazard model of the form

$$h_{T^{A_0=a}}(t)=\alpha_0^*(t)+\alpha_A(t)a. \tag{12}$$

It follows that we could use an additive hazard model for $\Pr(Y_1^{a_0=a}=0|L_0)$ in the sequential trials analysis and an additive hazard model for $\Pr(Y_1^{a_0=a}=0)$ in the MSM-IPTW analysis, and that both models can be correctly specified.

Next consider the probabilities $\Pr(Y_2^{\bar{a}_1=a}=0)=\Pr(Y_2^{\bar{a}_1=a}=0|Y_1^{a_0=a}=0)\Pr(Y_1^{a_0=a}=0)$ and $\Pr(Y_2^{\bar{a}_1=a}=0|L_0)=\Pr(Y_2^{\bar{a}_1=a}=0|Y_1^{a_0=a}=0,L_0)\Pr(Y_1^{a_0=a}=0|L_0)$. Under the fully conditional additive hazard model in (9) it can be shown that (see below)

$$\Pr(Y_2^{\bar{a}_1=a}=0|Y_1^{a_0=a}=0,L_0)=\exp\left\{-\int_1^2(\alpha_0(u)+\alpha_A(u)a)du+\Delta(a,L_0)\right\} \tag{13}$$

and

$$\Pr(Y_2^{\bar{a}_1=a}=0|Y_1^{A_0=a}=0)=\exp\left\{-\int_1^2(\alpha_0(u)+\alpha_A(u)a)du+\Delta^*(a)\right\}, \quad (14)$$

where

$$\Delta(a,L_0)=\log\left\{\Pr(L_1=0|Y_1=0,A_0=a,L_0)+e^{-\int_1^2\alpha_L(u)du}\Pr(L_1=1|Y_1=0,A_0=a,L_0)\right\}$$

and

$$\Delta^*(a)=\log\left\{\frac{e^{\Delta(a,0)}\Pr(L_0=0)+e^{\Delta(a,0)-\int_0^1\alpha_L(u)du}\Pr(L_1=1)}{\Pr(L_0=0)+e^{-\int_0^1\alpha_L(u)du}\Pr(L_1=1)}\right\}.$$

It follows that there exists an underlying conditional hazard model that gives rise to an additive model for the sequential trials analysis and for the MSM-IPTW analysis. To obtain expressions (13) and (14) we used the result that the probability $\Pr(Y_2^{\bar{a}_1=a}=0|Y_1^{a_0=a}=0)$ can be written

$$\Pr(Y_2^{\bar{a}_1=a}=0|Y_1^{a_0=a}=0)=\sum_{l_0}\Pr(Y_2^{\bar{a}_1=a}=0|Y_1^{a_0=a}=0,L_0=l_0)\frac{\Pr(Y_1=0|A_0=a,L_0=l_0)\Pr(L_0=l_0)}{\sum_{l_0'}\Pr(Y_1=0|A_0=a,L_0=l_0')\Pr(L_0=l_0')} \quad (15)$$

and the first term in the sum can then be written

$$\Pr(Y_2^{\bar{a}_1=a}=0|Y_1^{a_0=a}=0,L_0)=\sum_{l_1}\Pr(Y_2=0|Y_1=0,\bar{A}_1=a,L_0,L_1=l_1)\Pr(L_1=l_1|Y_1=0,A_0=a,L_0). \quad (16)$$

Secondly consider a fully conditional Cox proportional hazards model of the form

$$h(t|\bar{A}_{\lfloor t\rfloor},\bar{L}_{\lfloor t\rfloor})=h_0(t)e^{\beta_A A_{\lfloor t\rfloor}+\beta_L L_{\lfloor t\rfloor}}. \quad (17)$$

Under this model the conditional survival probability at time 1 is $\Pr(Y_1=0|A_0,L_0)=\exp\left(-H_0(1)e^{\beta_A A_0+\beta_L L_0}\right)$ where $H_0(1)=\int_0^1 h_0(u)du$. Using this, the marginal probability of interest can be written

$$\Pr(Y_1^{a_0=a}=0)=\exp\left(-H_0(1)e^{\beta_A A_0}\right)\Pr(L_0=0)+\exp\left(-H_0(1)e^{\beta_A A_0+\beta_L}\right)\Pr(L_0=1). \quad (18)$$

This expression for $\Pr(Y_1^{a_0=a}=0)$ is not the form of the survival probability from a marginal Cox proportional hazards model. Therefore, if a Cox model was assumed for $h_{T^{\underline{a}_0}}(t|L_0)$ in the sequential trials analysis, then this does not imply a Cox model for $h_{T^{\underline{a}_0}}(t)$ in the MSM-IPTW analysis, and vice-versa. If Cox models are used for a sequential trials analysis and a MSM-IPTW analysis then the two analyses make different modelling assumptions that cannot both be true. In practice, however, a Cox model could be a good working model for both approaches.

The result in this section has implications for comparing estimates obtained from the MSM-IPTW approach and the sequential trials approach, and we make use of these in the simulation study in Section 6 of the main text by using additive hazards models.

References

Cole, S. and Hernán, M. (2008). Constructing inverse probability weights for marginal structural models. *American Journal of Epidemiology* **168,** 656–664.

Table 1: Simulation results. Summary of number of individuals observed at each time, number always treated and never treated, and corresponding percentages. Numbers and percentages are the mean across the 1000 simulations.

		MSM-IPTW Time k					Sequential trials Time k				
		0	1	2	3	4	0	1	2	3	4
**Scenario 1**											
Observed at time k	N	1000	829	694	588	502	2264	1414	873	509	238
	%	100	83	69	59	50	100	62	39	22	10
Always treated from time 0 to k	N	279	237	204	177	155	643	514	397	282	155
	%	28	29	29	30	31	28	36	46	55	65
Never treated from time 0 to k	N	721	424	249	144	82	1621	900	475	227	82
	%	72	51	36	25	16	72	64	54	45	35
**Scenario 2**											
Observed at time k	N	1000	821	675	556	458	3180	2178	1403	806	349
	%	100	82	68	56	46	100	68	44	25	11
Always treated from time 0 to k	N	53	45	39	34	29	197	142	98	61	29
	%	5	5	6	6	6	6	7	7	8	8
Never treated from time 0 to k	N	947	731	560	425	319	2983	2036	1305	745	319
	%	95	89	83	76	70	94	93	93	92	92
**Scenario 3**											
Observed at time k	N	1000	832	701	596	510	2083	1333	865	543	287
	%	100	83	70	60	51	100	64	42	26	14
Always treated from time 0 to k	N	387	326	278	241	210	700	563	446	337	210
	%	39	39	40	40	41	34	42	52	62	73
Never treated from time 0 to k	N	613	352	213	129	77	1383	770	419	206	77
	%	61	42	30	22	15	66	58	48	38	27

Figure 1: Simulation results: bias in estimation of the risk difference using the sequential trials analysis and the MSM-IPTW analysis. The black line shows the bias at each time point and the grey area shows the Monte-Carlo 95% CI at each time point. The MSM-IPTW results are from the MSM not conditional on L_0.

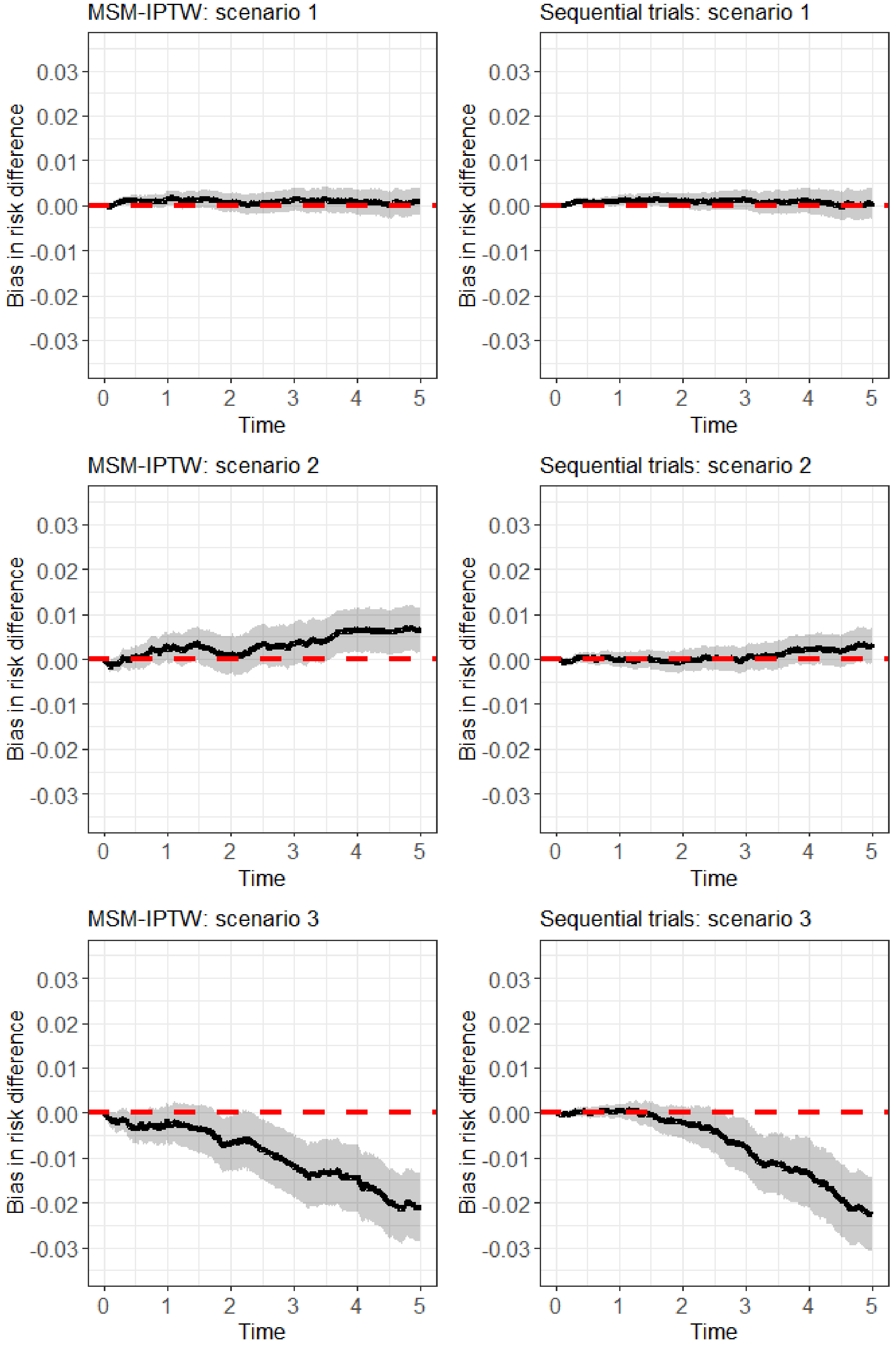

Figure 2: Simulation results: relative efficiency of the sequential trials analysis compared with the MSM-IPTW analysis, defined as the ratio of the empirical variances of the risk difference estimates at each time. The MSM-IPTW results are from the MSM not conditional on L_0.

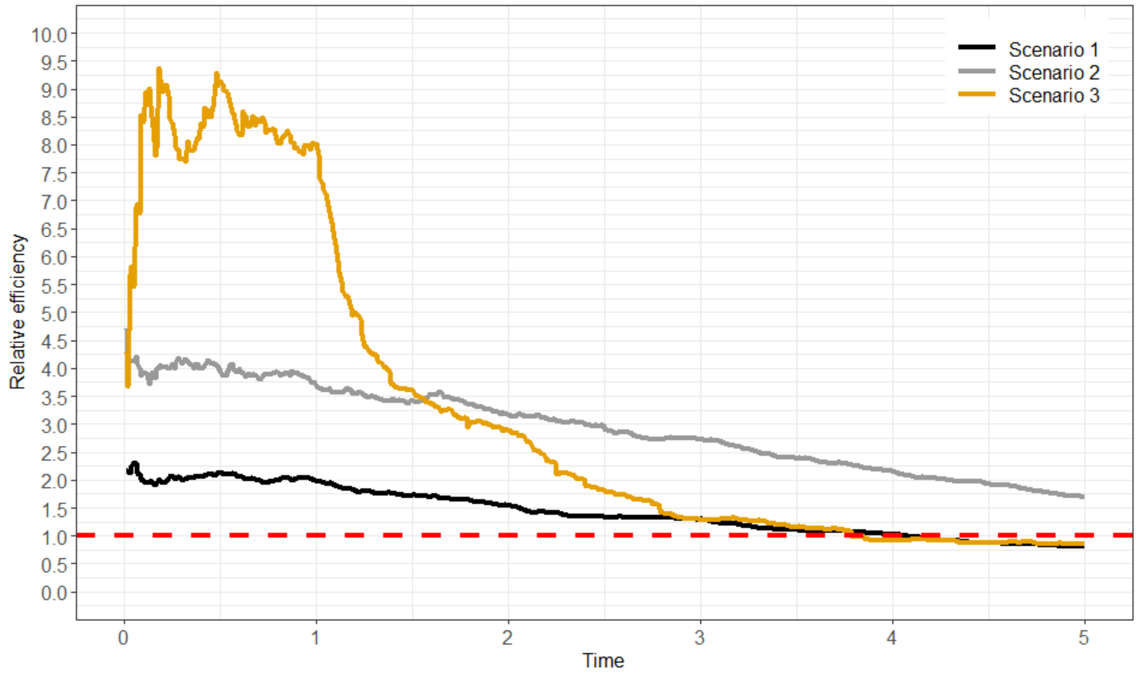

Daniel, R., Cousens, S., De Stavola, B., Kenward, M., and Sterne, J. (2013). Methods for dealing with time-dependent confounding. *Statistics in Medicine* **32,** 1584–1618.

Keogh, R., Seaman, S., Gran, J., and Vansteelandt, S. (2021). Simulating longitudinal data from marginal structural models using the additive hazard model. *Biometrical Journal* **63,** 1526–1541.

Martinussen, T. and Vansteelandt, S. (2013). On collapsibility and confounding bias in cox and aalen regression models. *Lifetime data analysis* **19,** 279–296.

Figure 3: Simulation results using truncated weights: bias in estimation of the risk difference using the sequential trials analysis and the MSM-IPTW analysis. The black line shows the bias at each time point and the grey area shows the Monte-Carlo 95% CI at each time point. The MSM-IPTW results are from the MSM conditional on L_0.

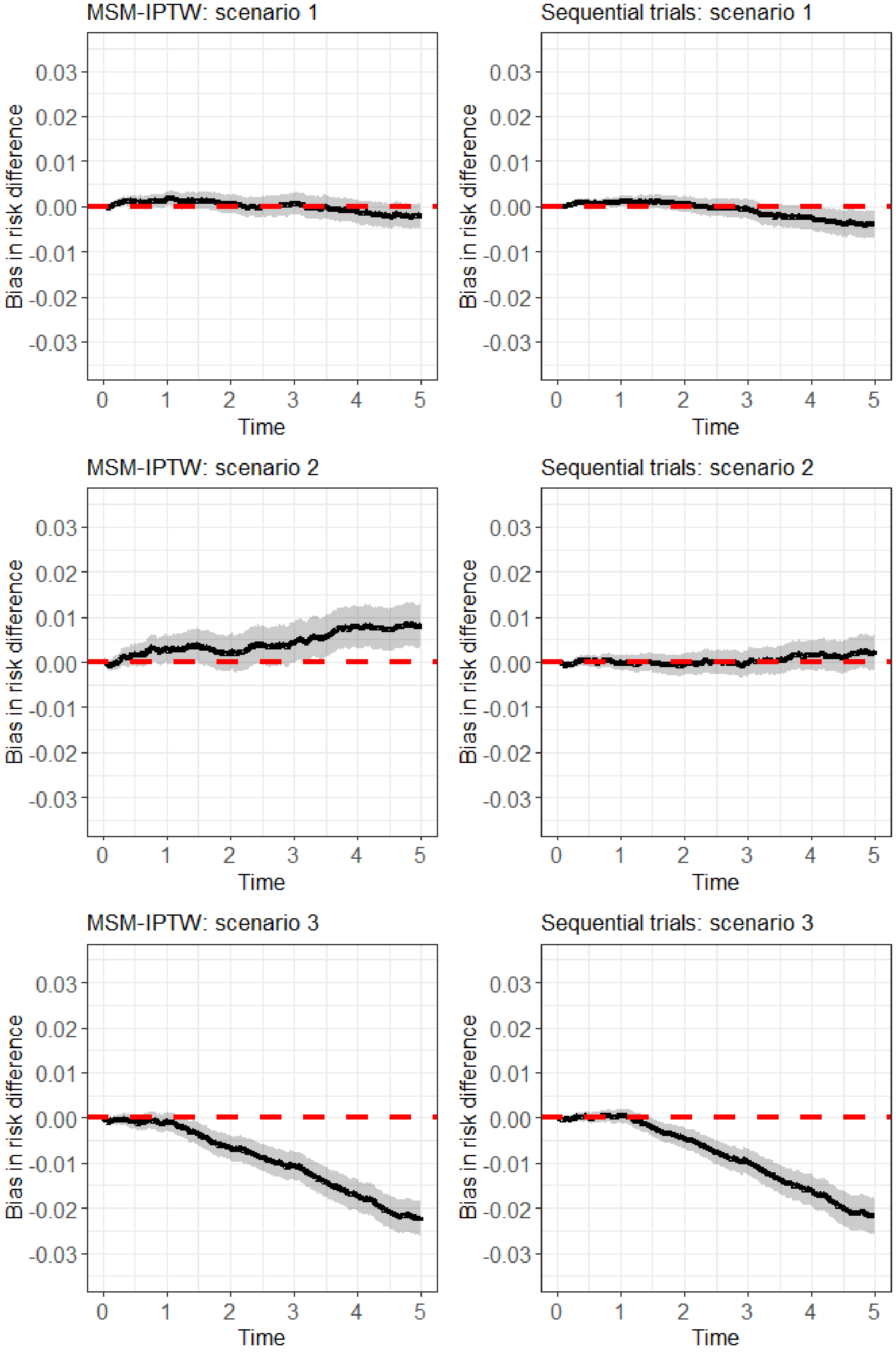

.

Figure 4: Simulation results using truncated weights: relative efficiency of the sequential trials analysis compared with the MSM-IPTW analysis, defined as the ratio of the empirical variances of the risk difference estimates at each time. The MSM-IPTW results are from the MSM conditional on L_0.

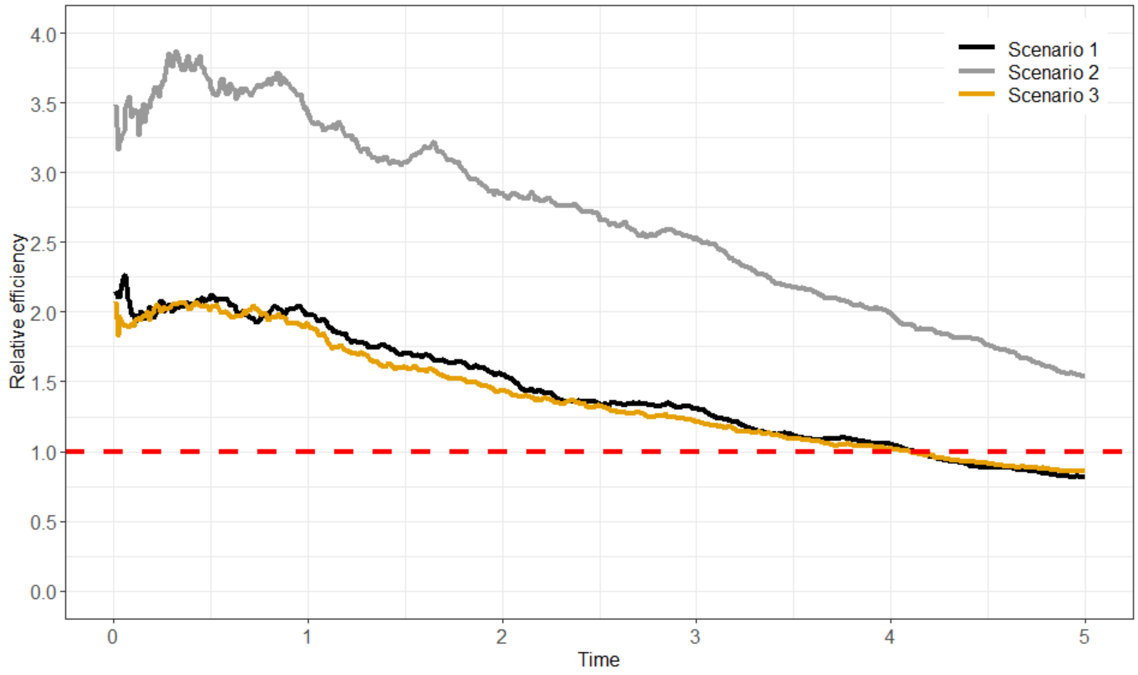

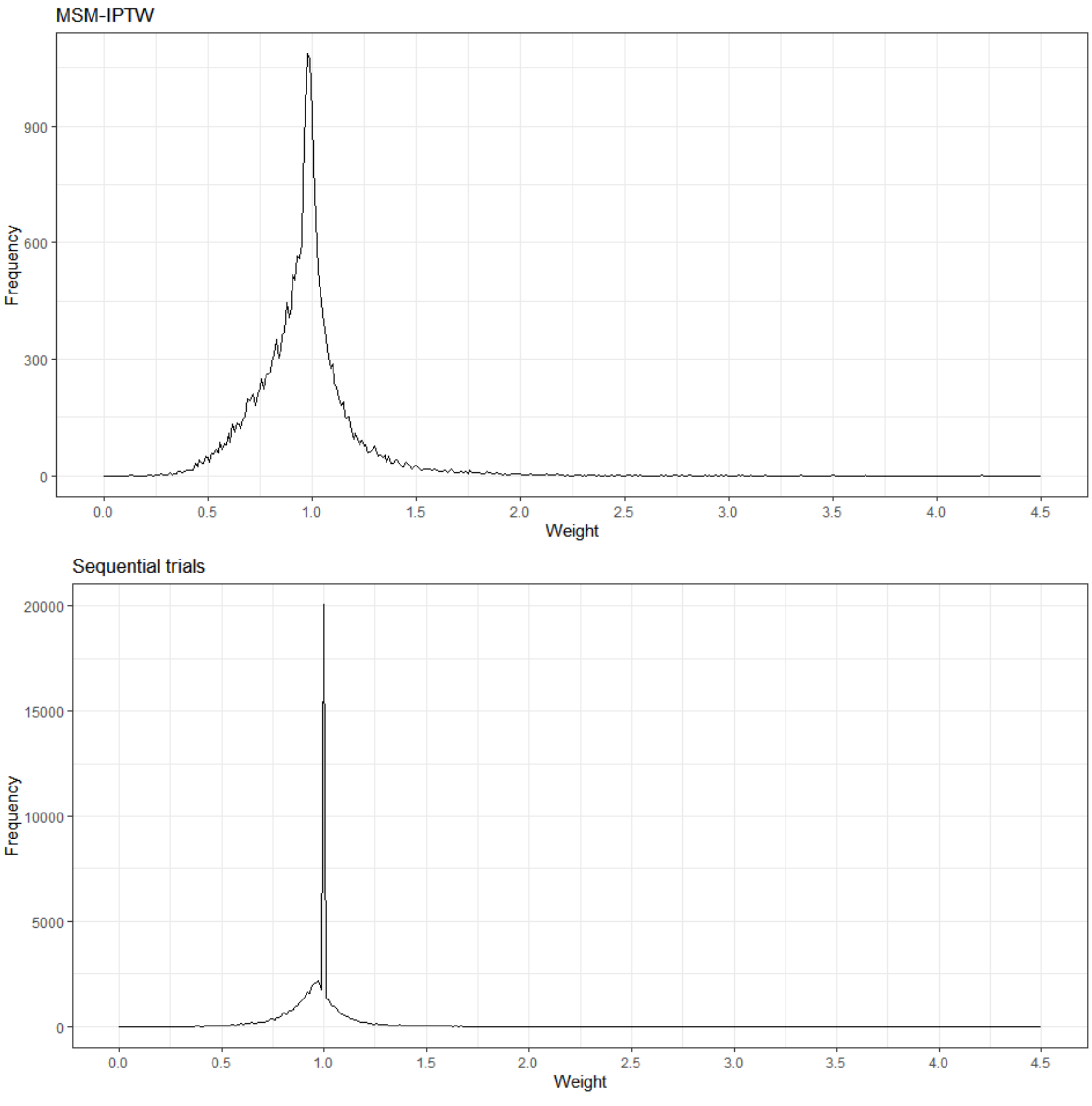

Figure 5: Plots showing the distribution of the weights used in the MSM-IPTW and sequential trials analyses of the application using UK CF Registry data.
